# Superconducting YAu_3_Si and Antiferromagnetic GdAu_3_Si with an Interpenetrating Framework Structure Built from 16-Atom Polyhedra

**DOI:** 10.1021/acs.inorgchem.1c03456

**Published:** 2022-02-28

**Authors:** Girma Hailu Gebresenbut, Lars Eriksson, Ulrich Häussermann, Andreas Rydh, Roland Mathieu, Olga Yu. Vekilova, Takayuki Shiino

**Affiliations:** † Department of Chemistry-Ångström Laboratory, Uppsala University, 751 21 Uppsala, Sweden; ‡ Department of Materials and Environmental Chemistry, Stockholm University, 106 91 Stockholm, Sweden; § Department of Physics, Stockholm University, 106 91 Stockholm, Sweden; ∥ Department of Materials Science and Engineering, Uppsala University, Box 35, 751 03 Uppsala, Sweden

## Abstract

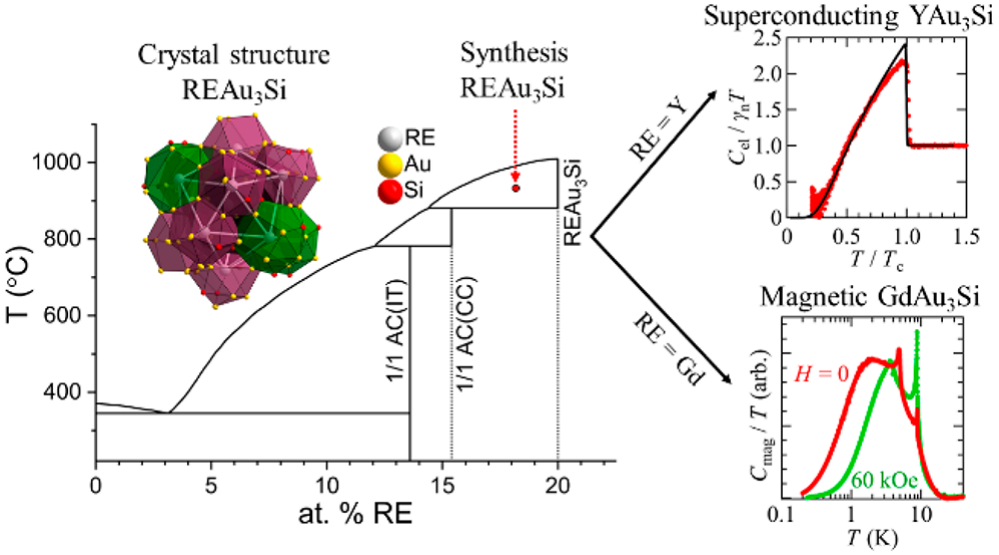

Investigations of reaction mixtures RE_*x*_(Au_0.79_Si_0.21_)_100–*x*_ (RE = Y and Gd) yielded the compounds REAu_3_Si which adopt a new structure type, referred to as GdAu_3_Si structure (*tP*80, *P*4_2_/*mnm*, *Z* = 16, *a* = 12.8244(6)/12.7702(2) Å, and *c* = 9.0883(8)/9.0456(2) Å for GdAu_3_Si/YAu_3_Si, respectively). REAu_3_Si was afforded as millimeter-sized faceted crystal specimens from solution growth employing melts with composition RE_18_(Au_0.79_Si_0.21_)_82_. In the GdAu_3_Si structure, the Au and Si atoms are strictly ordered and form a framework built of corner-connected, Si-centered, trigonal prismatic units SiAu_6_. RE atoms distribute on 3 crystallographically different sites and each attain a 16-atom coordination by 12 Au and 4 Si atoms. These 16-atom polyhedra commonly fill the space of the unit cell. The physical properties of REAu_3_Si were investigated by heat capacity, electrical resistivity, and magnetometry techniques and are discussed in the light of theoretical predictions. YAu_3_Si exhibits superconductivity around 1 K, whereas GdAu_3_Si shows a complex magnetic ordering, likely related to frustrated antiferromagnets exhibiting chiral spin textures. GdAu_3_Si-type phases with interesting magnetic and transport properties may exist in an extended range of ternary RE–Au–Si systems, similar to the compositionally adjacent cubic 1/1 approximants RE(Au,Si)_∼6_.

## Abstract 2

A novel structure type, *tP*80 and *P*4_2_/*mnm*, is discovered in two compounds: GdAu_3_Si and YAu_3_Si. The crystal structure was determined using SCXRD technique. Physical properties of both compounds were investigated experimentally by heat capacity, electrical resistivity, and magnetometry techniques and theoretically using the DFT method. While the GdAu_3_Si prevail complex magnetic ordering at the temperature around 10 K, YAu_3_Si becomes superconducting close to 1 K.

## 1 Introduction

Polar intermetallic compounds of gold with electropositive and post-transition metals/semimetals from groups 12–14 display peculiarities in their structural chemistry and physical properties, which has been attributed to the extraordinarily high electronegativity of gold (which is the highest among metallic elements) and associated relativistic effects in chemical bonding.^1^ The family of gold polar intermetallics is rather diverse and also includes icosahedral quasicrystals (iQCs), such as i-Na–Au–Ga, i-Ca–Au–Al(Ga)(In), i-RE–Au–Al (RE = Yb and Tm), i-RE–Au–Sn (RE = Ca and Yb), and a larger range of 1/1 cubic approximant crystal (AC) phases.^2−11^

The majority of gold-based iQCs are of Tsai-type^12^ and also contain rare-earth (RE) elements. These iQCs have attracted considerable attention because of expectations about unique physical properties associated to the quasiperiodic structure.^13,14^ Tsai-type iQCs are distinguished by their atomic cluster building unit (“Tsai-cluster”) consisting of four concentric shells and centered by a tetrahedral moiety.^15^ The radial dimension of a Tsai-cluster in gold-based iQCs is 15–16 Å. Their related 1/1 ACs are also built from Tsai-clusters and have similar chemical composition but are conventional (3D periodic) crystals.^16^ In both Tsai-type QCs and 1/1 ACs, RE atoms are arranged into icosahedra which represent one of the shells of a Tsai-cluster. AC phases play a pivotal role by providing local structural information (from standard crystallographic techniques) needed to determine the structures of iQCs and by providing references for physical properties of 3D periodic systems. Since the stability of Tsai-type QCs is linked to a very narrow valence electron per atom ratio, 1/1 ACs are found much more frequently than QCs.^17^ There are many phase diagrams (i.e., RE–Au–Si and RE–Au–Ge) for which hitherto only the 1/1 AC phase is known.

We recently reported that for Tsai-type 1/1 ACs in RE–Au–Si systems (RE; e.g., Gd, Tb, and Ho) the central tetrahedron of the Tsai clusters can be systematically replaced by a single RE atom, giving rise to a distinctly different variant of 1/1 AC phase with a composition RE_∼15.4_(Au,Si)_∼84.6_ instead of RE_∼13.6_(Au,Si)_∼86.4_.^18^ The regular ((Au,Si) tetrahedron centered) and RE-centered phase were termed AC(IT) and AC(CC), respectively. During the course of this study we discovered an even more RE-rich phase, which formed from the peritectic decomposition of AC(CC) at temperatures above 900 °C. In this paper, we report on the strictly Au,Si ordered structure and the physical properties of REAu_3_Si (RE = Y and Gd). With an RE content of 20 at. % the structure of REAu_3_Si significantly deviates from the Tsai-type cluster based and Au,Si disordered 1/1 AC structure, yet locally similarities are maintained (i.e., a 16-atom coordination environment for RE and icosahedral arrangement of RE atoms).

## 2 Methods

### 2.1 Synthesis

The starting materials were granules of the elements Gd and Au (Chempur 99.99%), Y (Chempur 99.9%), and Si (Highways International 99.999%). Prior the synthesis reactions, Au and Si were arc-melted in a ratio 79:21 (at. %) corresponding to the eutectic composition in the Au–Si phase diagram. The arc-melting procedure was repeated five-times to get homogeneous ingot. Actual reaction mixtures then constituted compositions Gd_*x*_(Au_0.79_Si_0.21_)_100–*x*_ with *x* in the range of 15–18. Reaction mixtures were investigated with differential scanning calorimetry (DSC) prior to solution-growth synthesis to extract liquid temperatures. Synthesis reactions targeting REAu_3_Si were carried out in alumina (Al_2_O_3_) crucibles from LSP Industrial Ceramics (USA), in the form of “Canfield Crucible Sets (CCS)”. The CCS consists of two flat-bottomed cylindrical crucibles and an alumina frit-disc with holes of ∼0.7–1 mm in diameter designed to separate solid grains from the liquid melt during centrifugation.^19^ A total mass of about 3 g was weighed inside a glovebox (Ar-atmosphere, <0.1 ppm O_2_) and loaded into the CCS, which was then encapsulated inside a stainless-steel ampule. Ampules were heated in a commercial multistep programmable muffle furnace to 1050 °C over a period of 10 h and dwelled for 3 h to ensure a homogeneous melt. Subsequently, the temperature was lowered to 920 °C using a cooling rate of 1 °C/h, and reactions were terminated by isothermally centrifuging off excess melt at the target temperatures.

### 2.2 Phase and Structure Analysis

The samples were studied with powder X-ray diffraction (PXRD), single-crystal X-ray diffraction (SCXRD), DSC, scanning electron microscopy (SEM) coupled with energy-dispersive X-ray spectroscopy (EDX), and magnetic property measurements. A Bruker D8 powder diffractometer with θ–2θ diffraction geometry and a Cu Kα radiation (Kα_1_ = 1.540598 Å and Kα_2_ = 1.544390 Å) was used for collecting PXRD intensities at room temperature. PXRD data were analyzed with the HighScore Plus 3.0 software from PANalytical^20^ and the Fullprof Suite.^21^ Powdered samples were applied to a zero-diffraction plate, and diffraction patterns were measured in a 2θ range of 7–90°. A Bruker D8 VENTURE single-crystal X-ray diffractometer with Mo Kα radiation (Kα = 0.71073 Å) with Incoatec microfocus source (IμS 3.0) and Photon II CCD area detector was utilized to collect SCXRD intensities at room temperature. Diffraction data covering a half (full) sphere in reciprocal space were collected with 100% completeness. SCXRD data reduction and numerical absorption corrections were carried out using the APEX III software from Bruker.^22^ The crystal structure was solved and refined using the software SHELXT^23^ and JANA2006,^24^ respectively. The structures were visualized using Diamond 3.2K4.^25^ Electron densities were visualized using the software VESTA.^26^ DSC measurements were carried out with a NETZSCH STA 449 F1 Jupiter instrument. Sample specimens (typically faceted grains, REAu_3_Si) with total mass of ∼50 mg were placed in polycrystalline sapphire crucibles (OD = 5 mm, ID = 4 mm) and a heating/cooling cycle to 1150 °C was carried out at a rate of 10 °C/minute under an Ar flow of ∼40 mL/min. An empty crucible served as reference. The crucibles used for the actual DSC measurement were first carried through identical heating/cooling protocol to the sample and the data were used as background. Scanning electron microscopy (SEM) investigations employed a Zeiss–Merlin instrument equipped with energy-dispersive X-ray (EDX) spectroscopy for elemental analysis with X-Max 80 mm^2^ Silicon Drift Detector with high sensitivity and high count rates. Prior to the SEM/EDX experiments samples were cross section polished gently for 20 h using Ar^+^-ion beam in a Cross-Section Polisher SM-09011 instrument from JEOL. EDX data was collected with an acceleration voltage of 20 kV over larger areas (∼100 × 100 μm^2^) on at least 20 points.

### 2.3 Physical Property Measurements

(Polycrystalline) samples for physical property measurements were prepared from pieces of crushed specimens obtained from the solution-growth synthesis experiments. Direct current (dc) magnetization measurements (on ∼10 mg sample specimens) were carried out using an MPMS XL SQUID magnetometer equipped with a superconducting magnet (up to ±50 kOe) and a Physical Property Measurement System (PPMS) with a superconducting magnet (up to ±90 kOe), both from Quantum Design, Inc. Heat capacity measurements were carried out using a Bluefors dilution refrigerator equipped with a superconducting magnet (up to ±120 kOe). The heat capacity data was collected down to 100–200 mK on tiny sample fragments (with a volume of approximately 1 × 10^5^ μm^3^) using a differential membrane-based nanocalorimeter.^27^ We calibrated the specific-heat values in molar unit by multiplying the heat capacity data by a constant to match the model curve of γ*T* + *C*_D_ at high temperatures, where γ is the electronic specific heat coefficient (we determined γ = 1 mJ/K^2^ mol for GdAu_3_Si and YAu_3_Si) and *C*_D_ is the specific heat of the Debye model (we determined the Debye temperature θ_D_ = 170 K for GdAu_3_Si and θ_D_ = 193 K for YAu_3_Si); see also ref (28). The electrical resistivity (for sample specimens with dimension ∼0.2 × 0.25 × 1 mm^3^) was measured using the conventional four-probe method with the dilution refrigerator and the PPMS.

### 2.4 Theoretical Calculations

The ground state of GdAu_3_Si was studied theoretically using a first-principles density functional theory (DFT) approach within the projector-augmented wave (PAW) method^29^ as implemented in Vienna Ab Initio Simulation Package (VASP).^30−32^ The generalized gradient approximation in its Perdew-Burke-Erzernhof flavor for exchange and correlation potential and energy was used.^33^ All simulations of magnetic properties were done using the Molecular Dynamics Monte Carlo (MDMC) method.^34^

Standard approach at 0 K is to compare known magnetic orderings (for example, FM and simple AFM) and choose the state with the lowest total energy. In the case of complex magnetism, like different ferrimagnetic states, such an approach easily misleads to a wrong ground-state magnetic structure. The MDMC method^34^ solves this issue. It finds the proper magnetic ordering in the course of standard first-principles calculations. At 0 K, the method is based on a Monte Carlo (MC) technique and efficiently explores the whole phase space of magnetic structures. At higher temperatures, the method takes care of coupling between spins and atomic vibrations via AIMD, and in particular can treat non-Heisenberg systems. Only collinear 0 K MDMC simulations were carried out in this work. Further, the obtained magnetic structures were tested by the noncollinear version of VASP and found to be collinear. All calculations referred to the 80 atom unit cell of magnetic GdAu_3_Si. The structures were relaxed to *p* = 0 GPa with the accuracy of few kbar. A 2 × 2 × 2 *k*-point Monkhorst–Pack grid^35^ was used for all integrations over the Brillouin zone. LDA+U approximation in Dudarev’s formulation^36^ with *U* = 6 eV was applied on the f-electron states of Gd.^37^ All the calculations were done at temperature *T* = 0 K. The energy cutoff for plane waves was set to 400 eV.

## 3 Results and Discussion

### 3.1 Partial Pseudobinary RE–(Au_0.79_Si_0.21_) Systems and the Phase REAu_3_Si

**Figure 1:**
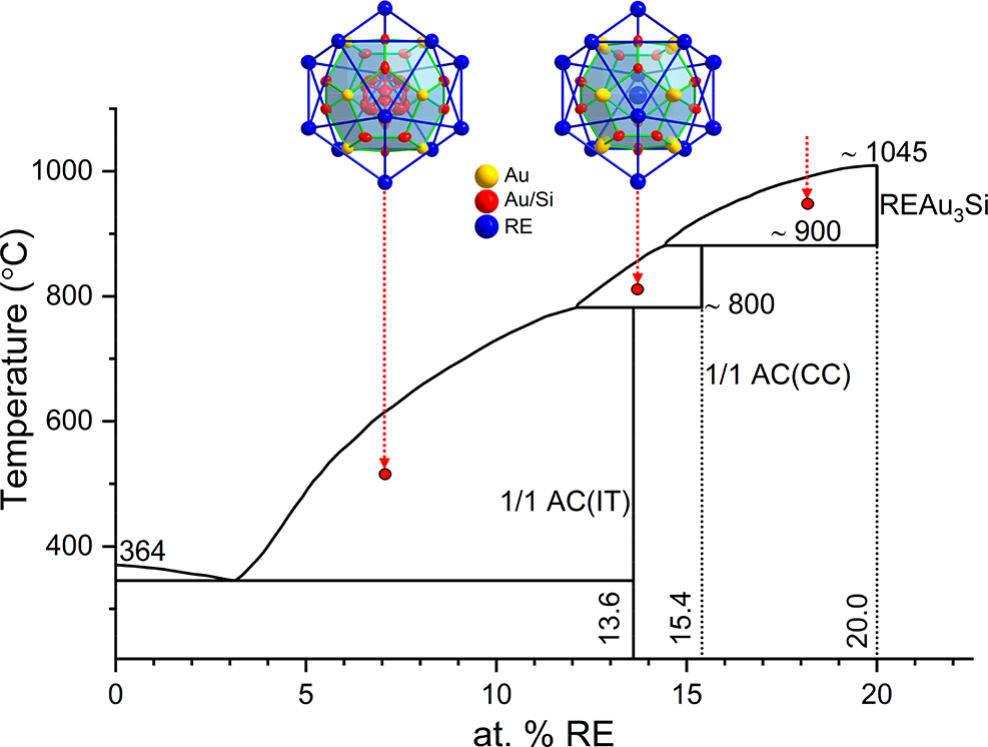
Sketch of the pseudobinary RE–(Au_0.79_Si_0.21_) partial phase diagram. The inset shows the two innermost shells of the Tsai cluster ((Au,Si) dodecahedron and RE icosahedron) which are centered with an orientationally disordered (Au,Si) tetrahedron (for AC(IT), left) and a single RE atom (for AC(CC), right), respectively; the red arrows indicate typical synthesis paths which were followed to prepare 1/1 AC(IT), 1/1 AC(CC), and REAu_3_Si phases.

The deep eutectic point in the Au–Si phase diagram for Au_0.79_Si_0.21_ (∼364 °C) has been previously exploited for the investigation of the (Au–Si)-rich part of ternary RE–Au–Si systems, considering these systems as pseudobinary RE_*x*_(Au_0.79_Si_0.21_)_100–*x*_ for *x* up to 15.^18,28^ In these investigations melts with *x* = 4–14 were slowly cooled over the liquidus which allows crystallization of the phases most rich in (Au–Si) below their peritectic decomposition temperatures (Figure 1). The solution growth experiments were terminated by centrifuging off isothermally excess liquid. High-melt crystallization typically affords ultrapure, single-crystalline products.^38^ For *x* up to 10–11 they corresponded to regular 1/1 AC phase with an orientationally disordered (Au,Si)_4_ tetrahedron at the Tsai-cluster center ((AC(IT)) cf. inset in Figure 1). For higher *x* (12–13), a variant of the 1/1 AC phase with a single RE atom at the cluster center was found (AC(CC)).^18,28^ In this case, centrifugation temperatures above 800 °C had to be employed, and it is not (yet) clear whether the AC(CC)-phases are thermodynamically stable at low temperatures or represent high-temperature phases. The RE content in the AC(CC) phases is significantly increased (which has profound consequences to their magnetic properties^18^). However, the Au/Si composition of the AC(IT) and AC(CC) phases, RE_13.6_(Au_∼0.82_Si_∼0.18_)_86.4_ and RE_15.4_(Au_∼0.81_Si_∼19_)_84.6_, respectively, are very close to nominally employed Au_0.79_Si_0.21_ which justifies the pseudobinary approach.

**Figure 2:**
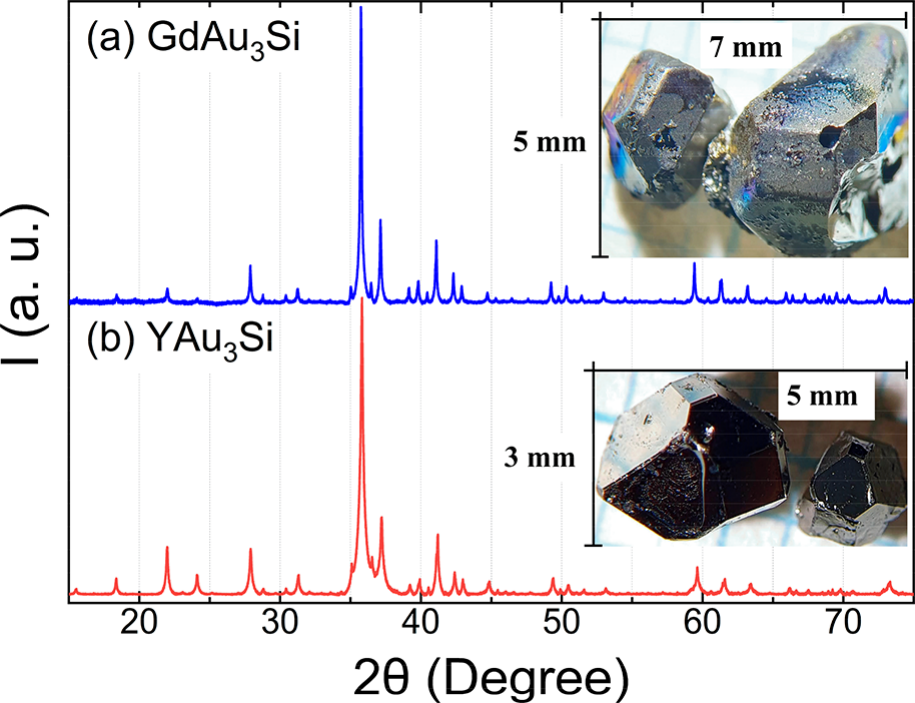
PXRD patterns for (a) GdAu_3_Si and (b) YAu_3_Si. X-ray scattering background and diffraction peaks from Cu Kα_2_ radiation have been removed from each pattern for clarity. The inset photograph shows isolated grains from each sample.

AC(CC) phases undergo peritectic decomposition at around 900 °C into melt and a phase even more rich in RE.^18,28^ This observation stimulated the extension of solution-growth experiments to the more RE-rich side for which melts with *x* ≈ 18 and centrifugation temperatures around 920 °C were employed. For this study, RE = Y and Gd were chosen. As typical of this method, large (millmeter-sized) and facetted crystal specimens could be isolated (inset in Figure 2). EDX analyses of the synthesis products resulted in Gd_21.1(2)_Au_60.9(2)_Si_18.0(1)_ and Y_19.7(1)_Au_62.5(1)_Si_17.8(1)_; see Figure S1. This pointed strongly to a stoichiometric composition REAu_3_Si and indicates that with increasing RE content (*x* > 17–18) the phase diagram cannot be considered anymore as pseudobinary RE_*x*_(Au_0.79_Si_0.21_)_100–*x*_. Figure 2 shows the PXRD patterns for GdAu_3_Si and YAu_3_Si. These are very similar and can be indexed to a primitive tetragonal lattice with *a* ≈ 12.8 Å and *c* ≈ 9.05 Å. The whole diffraction profile fitting of the patterns, using the structure model obtained from SCXRD refinement, is shown in Figure S2.

**Figure 3:**
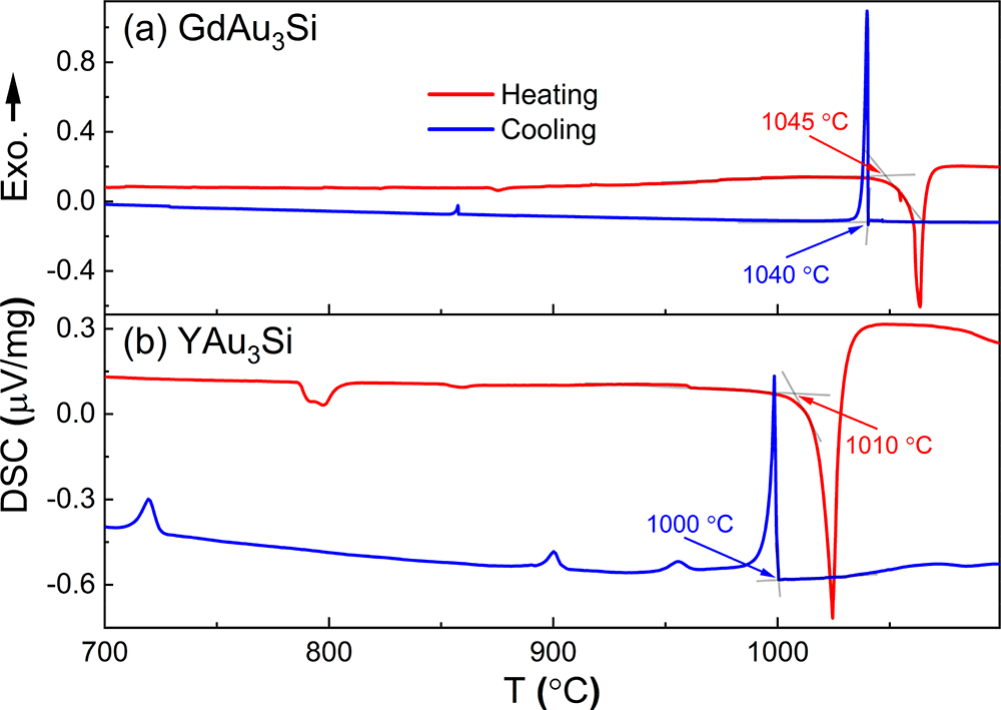
DSC traces for (a) GdAu_3_Si and (b) YAu_3_Si crystalline specimens. Temperatures of endothermic (decomposition) and exothermic (crystallization) events are estimated by extrapolation.

Figure 3 shows the DSC traces for GdAu_3_Si and YAu_3_Si upon a heating and cooling cycle. The small event at 870/850 °C (heating/cooling) for GdAu_3_Si is attributed to melting/crystallization of a small amount of residual flux with the composition Au_∼0.9_Si_∼0.1_ on the surface of the employed crystal specimen. The event at 1045/1040 °C indicates the liquid/solid temperature for the composition Gd_20_Au_60_Si_20_ and most likely corresponds to congruent melting/solidification of GdAu_3_Si, since the sample specimen after the DSC cycle appeared spherical and the PXRD pattern was virtually unchanged. In contrast, the heating trace of YAu_3_Si shows several smaller but distinguished endothermic events before the pronounced event indicating formation of liquid phase (at around 1000 °C). An annealing experiment at 875 °C (after the first endothermic event) produced a mixture of YAu_3_Si and AC(CC) phase (see Figures S3). Thus, the thermal behavior of YAu_3_Si remains unclear. It may be suspected that this compound represents a (metastable) high-temperature phase and that the exothermic conversion into the thermodynamic ground state at temperatures below 700 °C cannot be detected in DSC experiments because of a slow kinetics.

### 3.2 Crystal Structure of GdAu_3_Si and YAu_3_Si

The crystal structure of REAu_3_Si was determined from single-crystal X-ray diffraction data. Refinement results are shown in Table 1 and independent atomic positions and displacement parameters are listed in Table 2. The primitive tetragonal unit cell (space group *P*4_2_/*mnm* (#136)) contains 80 atoms which are distributed over 9 crystallographically independent sites: 5 Au, 3 Gd, and 1 Si. There is no detectable chemical (Au/Si) disorder, which is characteristic for the 1/1 AC systems. However, unlike in the GdAu_3_Si compound, an 8*i* Au position was found positionally disordered in YAu_3_Si (see Figure S4). Hence, the position was modeled as a mutually exclusive Au3/Au3′ split position with ∼0.93/0.07 occupancies, respectively. Furthermore, the atomic displacement parameters for YAu_3_Si are about twice that for GdAu_3_Si. This and the positional disorder may also be indicating a room temperature metastable nature of YAu_3_Si. The following description of the structure will be based on the parameters for GdAu_3_Si. Table 3 lists relevant interatomic distances (an extended distance table is given in Table S1).

**Table 1:** SCXRD Refinement and EDX Results for REAu_3_Si (RE = Gd and Y)

parameters	GdAu_3_Si	YAu_3_Si
empirical formula	GdAu_3_Si	YAu_3_Si
refined composition (at. %)	Gd_20_Au_60_Si_20_	Y_20_Au_60_Si_20_
EDX (at. %)	Gd_21.1(2)_Au_60.9(2)_Si_18.0(1)_	Y_19.7(1)_Au_62.5(1)_Si_17.8(1)_
formula weight	1552.5	1415.8
temperature/K	293	293
crystal system	tetragonal	tetragonal
space group	*P*4_2_/*mnm*	*P*4_2_/*mnm*
*a*/Å	12.8244(6)	12.7702(2)
*c*/Å	9.0883(8)	9.0456(2)
volume/Å^3^	1494.7(2)	1475.14(5)
*Z*	16	16
ρ_calc_, g/cm^3^	13.7971	12.7492
μ/mm^–1^	134.993	134.615
*F*(000)	5040.0	4640.0
radiation	Mo Kα (λ = 0.71073)	Mo Kα (λ = 0.71073)
2θ range, data collection/deg	4.5–63.04	4.52–83.88
index ranges	–18 ≤ *h* ≤ 18, –18 ≤ *k* ≤ 18, –11 ≤ *l* ≤ 13	–24 ≤ *h* ≤ 23, –23 ≤ *k* ≤ 23, –16 ≤ *l* ≤ 16
reflections collected	15394	92034
ind. reflections [all data]	1349	2792^a^
ind. reflections [*I* ≥ 3σ(*I*)]	1283	2283^a^
merging *R* indices	*R*_int_ = 0.0267, *R*_sigma_ = 0.0266	*R*_int_ = 0.0572, *R*_sigma_ = 0.0510
constraint/restraint/parameter	0/0/114	19/0/174^a^
goodness-of-fit [all data]	2.12^b^	1.73^b^
goodness-of-fit [*I* ≥ 3σ(*I*)]	2.13^b^	1.82^b^
final *R* indexes [*I* ≥ 3σ(*I*)]	*R*_1_ = 0.0183, *wR*_2_ = 0.0469	*R*_1_ = 0.0249, *wR*_2_ = 0.0471
final *R* indexes [all data]	*R*_1_ = 0.0204, *wR*_2_ = 0.0479	*R*_1_ = 0.0373, *wR*_2_ = 0.0497
largest diff. peak/hole/e Å^–3^	3.89/–1.62	4.63/–3.59

a Due to structural (positional) disorder in YAu_3_Si, the data collection strategy was extended to cover a full-sphere in reciprocal space with narrow step widths and longer exposure times; more parameters were refined.

b The relatively higher GOF values come partly from using the program Jana2006.

**Table 2:** Atomic Coordinates and Equivalent Atomic Displacement Parameters (*U*_eq_) of Independent Atomic Positions for REAu_3_Si (RE = Gd and Y) Obtained from SCXRD Refinement^a^

atom	Wyck.	S.O.F.	*x*/*a*	*y*/*b*	*z*/*c*	*U*_eq._ [Å^2^]
GdAu_3_Si						
Gd1	4*e*	1	1/2	1/2	0.2169(2)	0.0105(2)
Gd2	4*f*	1	0.22224(8)	0.22224(8)	0	0.0163(3)
Gd3	8*i*	1	0.63183(8)	0.11657(8)	0	0.0109(2)
Au1	8*i*	1	0.46064(6)	0.30558(7)	0	0.0130(2)
Au2	8*j*	1	0.32172(4)	0.32172(4)	0.7248(1)	0.0142(1)
Au3	8*i*	1	0.49648(7)	0.34779(6)	1/2	0.0162(2)
Au4	8*j*	1	0.66983(5)	0.33017(5)	0.8422(1)	0.0153(1)
Au5	16*k*	1	0.41023(5)	0.10204(5)	0.84455(6)	0.0163(1)
Si1	16*k*	1	0.5118(3)	0.2552(3)	0.7334(5)	0.0150(9)
YAu_3_Si						
Y1	4*e*	1	1/2	1/2	0.2168(3)	0.0253(9)
Y2	4*f*	1	0.2228(1)	0.2228(1)	0	0.0283(4)
Y3	8*i*	1	0.6317(1)	0.1166(1)	0	0.0208(3)
Au1	8*i*	1	0.46101(5)	0.30589(5)	0	0.0276(4)
Au2	8*j*	1	0.66978(4)	0.33022(4)	0.84199(9)	0.0322(4)
Au3	8*i*	0.933(5)	0.4985(2)	0.3464(2)	1/2	0.0331(9)
Au3′	8*i*	0.067(5)	0.474(1)	0.369(1)	1/2	0.0331(9)
Au4	8*j*	1	0.82152(4)	0.17848(4)	0.77523(9)	0.0293(4)
Au5	16*k*	1	0.41031(4)	0.10177(4)	0.84458(6)	0.0323(4)
Si1	16*k*	1	0.5114(2)	0.2546(2)	0.7346(3)	0.0241(6)

a Wyckhoff positions (Wyck.) and site occupancy factors (S.O.F.) are listed. *U*_eq._ = 1/3(*U*_11_ + *U*_22_ + *U*_33_).

**Table 3:** Relevant Interatomic Distances in the GdAu_3_Si Structure as Obtained from SCXRD Refinement

atom pair			*d*/Å (<3.5 Å)	atom pair			*d*/Å (<6 Å)
Gd1	Au4	2x	3.1266(7)	Gd1	Gd1	1x	3.942(2)
	Si1	4x	3.175(4)		Gd2	2x	4.782(1)
	Au1	4x	3.218(1)		Gd1	1x	5.146(2)
	Au3	4x	3.230(1)		Gd2	2x	5.409(1)
	Au2	2x	3.2765(6)		Gd3	4x	5.561(1)
Gd2	Au3	2x	3.031(1)		Gd3	4x	5.581(1)
	Au2	2x	3.084(1)	Gd2	Gd3	2x	4.731(2)
	Au5	4x	3.191(1)		Gd3	4x	5.1251(8)
	Au1	2x	3.239(1)		Gd3	2x	5.425(2)
	Au4	2x	3.252(1)	Gd3	Gd3	1x	4.514(2)
	Si1	4x	3.445(4)		Gd3	1x	4.563(2)
Gd3	Si1	2x	3.117(4)		Gd3	4x	5.5530(9)
	Au3	1x	3.126(1)				
	Au4	2x	3.130(1)				
	Au5	2x	3.1733(6)				
	Au5	2x	3.179(1)				
	Au5	2x	3.185(1)				
	Au1	1x	3.270(1)				
	Au2	2x	3.276(1)				
	Si1	2x	3.377(4)				
Si1	Au4	1x	2.452(2)				
	Au3	1x	2.476(2)				
	Au5	1x	2.509(2)				
	Au5	1x	2.530(2)				
	Au1	1x	2.567(2)				
	Au2	1x	2.588(2)				

**Figure 4:**
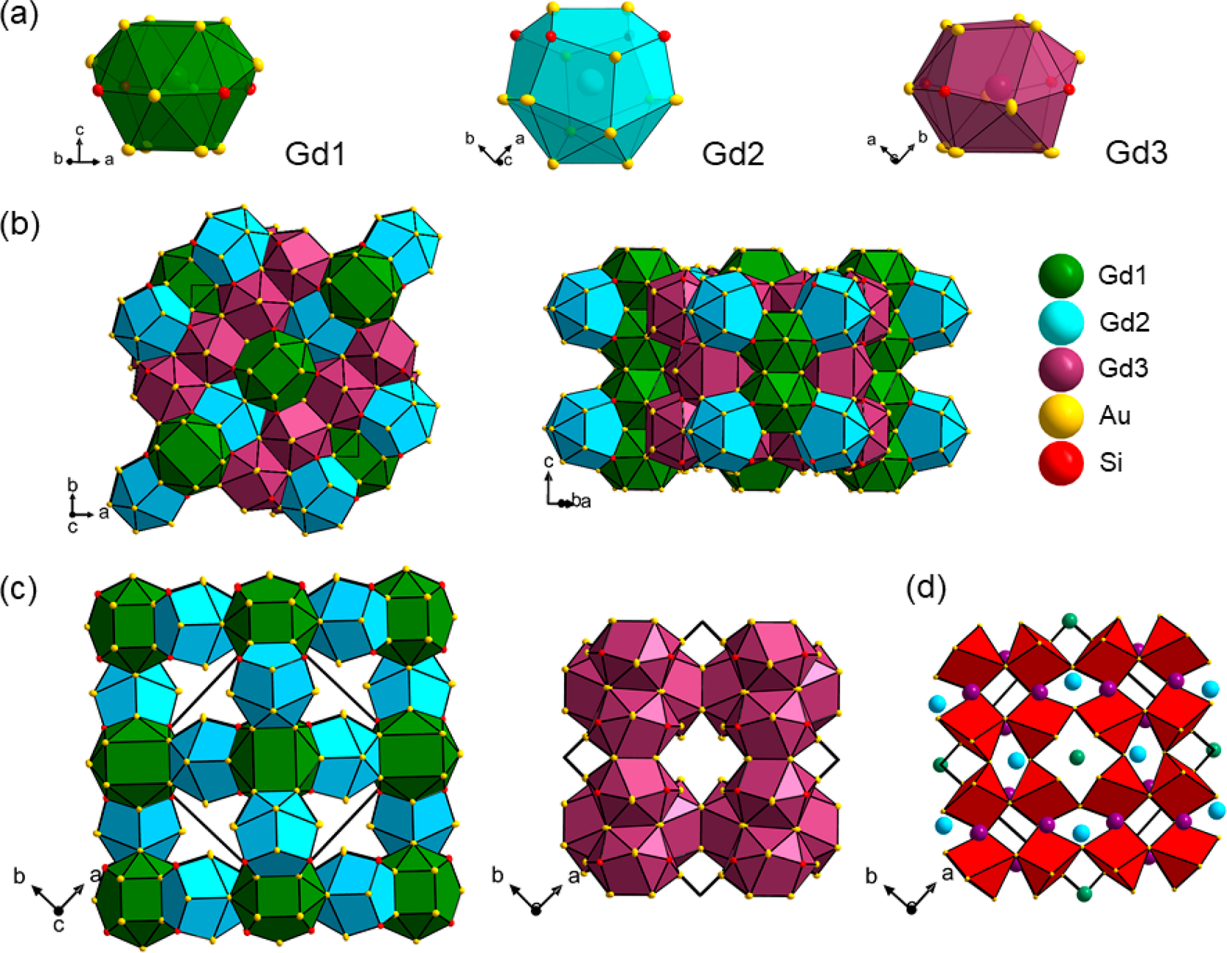
(a–c) Polyhedral crystal structure description for GdAu_3_Si. Au and Si atoms are presented as thermal ellipsoids at the 70% probability level. (d) Polyanionic Au_3_Si substructure corresponding to a framework of corner-connected Si-centered trigonal prims SiAu_6/2_.

The GdAu_3_Si structure may be described using the Gd coordination polyhedra which possess the local symmetries 4*e* (2*mm*), 4*f* (*m*2*m*), and 8*i*(*m*), as shown in Figure 4a. Each Gd atom is surrounded by 12 Au and 4 Si atoms, which provide a well-defined coordination shell with Gd–(Au,Si) distances in a range of 3.03–3.47 Å (cf. Table 3), clearly separated from next-nearest-neighbor distances starting off at 4.43 Å. The three kinds of polyhedra commonly provide space filling (Figure 4b). Their linkage can also be visualized as interpenetrating network (Figure 4c): Gd1 polyhedra are condensed into rows along the [001] direction via shared rectangular faces. These rows are linked by Gd2 polyhedra in the [110] direction to yield a primitive cubic arrangement with channels along (1/2, 0, *z*), which is the direction of the 4_2_ axes. This network is interpenetrated by the framework formed by Gd3 polyhedra. Gd3 polyhedra are clustered into rows (via the 4_2_ operations) along the *c*-direction and rows are connected via rectangular faces in the [110] direction. Si atoms are strictly coordinated by 6 Au atoms in a trigonal prismatic fashion (Si–Au distances are in a narrow range 2.45–2.59 Å, cf. Table 3), and the Au_3_Si partial structure corresponds to an array of corner connected SiAu_6/2_ trigonal prisms (Figure 4d). Considering REAu_3_Si as polar intermetallics, the Au_3_Si substructure bears a polyanionic character. In this picture, bonding between Au–Au and Au–Si atoms is of a strong covalent nature, whereas interactions between RE (RE^3+^) and Au/Si are essentially electrostatic. In the electronic density of states (DOS) of REAu_3_Si states near the Fermi level originate from Au–d states with minor contributions from Si and Gd orbitals (cf. Figure S5). This resembles strongly to other gold-rich polar intermetallics, such as RE_3_Au_7_Sn_3_, for which a pronounced polar intermetallic character has been proven from detailed bonding analyses.^39^

**Figure 5:**
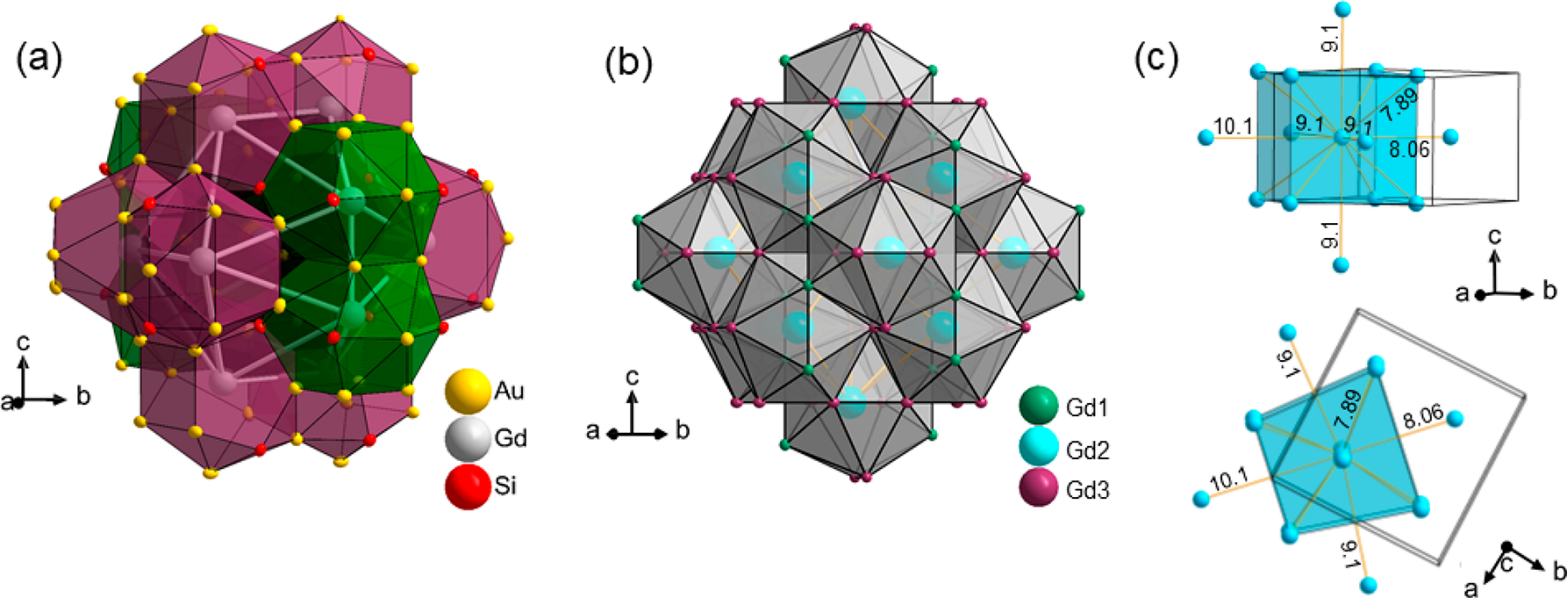
(a) Icosahedral environment of Gd2 by 4 Gd1 and 8 Gd3 atoms in the crystal structure of GdAu_3_Si. (b and c) The bcc-like arrangement of Gd2(Gd1,Gd3)_12_ icosahedra. (c) Only the icosahedra center (Gd2 atoms) are shown, and the Gd2–Gd2 distances are indicated.

Yet a different view of the GdAu_3_Si structure is provided when analyzing the Gd partial structure and identifying Gd2 atoms at the center of icosahedra formed by 4 Gd1 and 8 Gd3 atoms (Figure 5a). Gd2–Gd distances are in a range of 4.73–5.42 Å, and the edge lengths are between 3.94 and 5.42 Å (cf. Table 3). These icosahedral clusters in turn are arranged in a bcc-like (8 + 6) fashion (Figure 5b), where the nearest-neighbor centers (8) are 7.9 Å apart and the distance to the next-nearest-neighbor centers is on average 9.1 Å (Figure 5c). The icosahedral arrangement of Gd reminds of to the 1/1 AC structure, albeit icosahedra in the tetragonal GdAu_3_Si structure are more distorted and in addition appear more compressed (the center to corner distances of 1/1 AC icosahedra are in a range of 5.2–5.71 Å).^18^ Similar to the cubic 1/1 AC structure, the tetragonal GdAu_3_Si structure may represent a robust structure type that is realized for a larger number of REAu_3_Si and REAu_3_Ge compounds and thus could provide a playground for studying various physical properties and property changes when varying RE. In the following, we show superconductivity for YAu_3_Si and a peculiar magnetic behavior for GdAu_3_Si.

### 3.3 Superconducting YAu_3_Si

**Figure 6:**
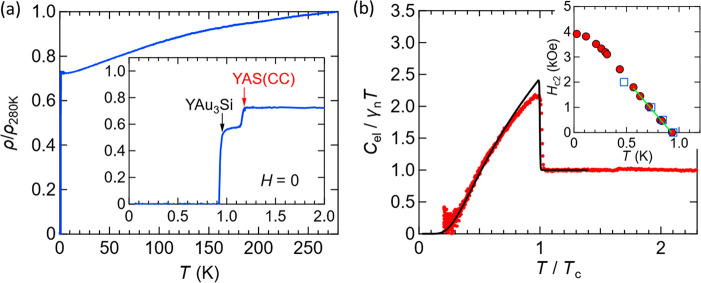
Superconductivity of YAu_3_Si. (a) Temperature dependence of the normalized electrical resistivity (ρ/ρ_280K_) under zero field; ρ_280K_ = 210 μΩ cm. The inset shows a close-up view for the resistivity near *T*_c_. (b) Plot of *C*_el_/γ_*n*_*T* as a function of *T*/*T*_c_. We set *T*_c_ = 0.97 K, which is slightly larger than the value (*T*_c_ = 0.94 K) determined from the resistivity data. The solid curve indicates the theoretical curve expected from the weak-coupling BCS model. The inset shows the upper critical field *H*_c2_ as a function of temperature. The filled-symbol points are obtained from the resistivity data, while the open-symbol points are from the specific heat data.

Figure 6a shows the temperature dependence of the electrical resistivity for YAu_3_Si. We observe a superconducting behavior at *T*_c_ = 0.94 K. The double-step behavior is attributed to a minor impurity of AC(CC) phase included in the YAu_3_Si grain which was used for this measurement. The Y–Au–Si AC(CC) phase has a slightly higher *T*_c_.^28^ The presence of tiny amounts of an AC(CC) impurity phase was also found for GdAu_3_Si, and the reason for this is not clear since solution grown sample specimens are typically single phase. The impurity phase cannot be detected in PXRD and SEM analyses or specific heat measurements. It expresses only in the resistivity and low-field magnetization data.

Figure 6b shows the normalized electronic specific heat divided by temperature (*C*_el_/γ_*n*_*T*) as a function of normalized temperature (*T*/*T*_c_), where *C*_el_ indicates the electronic contribution to the specific heat, and γ_*n*_ = 1.1 mJ/K^2^ mol is the electronic specific heat coefficient in the normal state. Note that we subtracted the phonon contribution (estimated from the *C*/*T* vs *T*^2^ plot) from the specific heat to estimate *C*_el_. This confirms the bulk nature of the superconductivity. The overall behavior is similar to the weak-coupling Bardeen–Cooper–Schrieffer (BCS) model, suggesting that the superconductivity of YAu_3_Si is of a conventional BCS type. We plot the upper critical field (*H*_c2_) versus temperature in the inset of Figure 6b. From the lowest temperature value of *H*_c2_, we estimate *H*_c2_(0) ≈ 3.9 kOe. From the data near *T*_c_, we obtain d*H*_c2_/d*T* = −4.86 kOe/K (cf. the solid line in the inset of Figure 6b), which allows us to estimate the orbital critical field at zero temperature using the Werthamer–Helfand–Hohenberg formula (in the dirty limit):^40^*H*_c2_^orb^(0) = −0.693*T*_c_(d*H*_c2_/d*T*) ≈ 3.15 kOe. The *H*_c2_^orb^(0) value is close to *H*_c2_(0); thus, the orbital effect mainly contributes to *H*_c2_ (rather than the spin paramagnetic effect).^40^ The estimated values of superconducting parameters are listed in Table 4. From the specific heat, we estimate the thermodynamic critical field *H*_c_ by calculating the condensation energy. The Ginzburg–Landau (GL) parameter κ at *T* = 0 is then estimated from the relation .^41,42^ Since , the superconductivity must be of type-II. See the caption of Table 4 for the other parameters. The overall superconducting behavior is similar to the Y–Au–Si AC(IT) and AC(CC) phases.^28^ See the Supporting Information (and ref 42 therein) for more detailed information regarding electrical resistivity data (see Figures S6 and S7) and specific heat data analysis (see Figures S8 and S9).

**Table 4:** Superconducting Parameters of YAu_3_Si^a^.

parameter	YAu_3_Si
*T*_c_ (K)	0.94
*H*_c2_(0) (kOe)	3.9
*H*_c_(0) (Oe)	69
*H*_c1_(0) (Oe)	4.5
κ	40
ξ(0) (nm)	29
λ(0) (μm)	1.2

a The lower critical field *H*_c1_ (*T* = 0) is estimated from the following relation , which is valid for large κ values.^41^ The coherence length *ξ*(*T* = 0) and penetration depth λ(*T* = 0) are estimated from the following relations: *H*_c2_(0) = Φ_0_/2π*ξ*(0)^2^ and κ ≡ λ/ξ where Φ_0_ is the magnetic flux quanta.

### 3.4 Antiferromagnetic GdAu_3_Si

**Figure 7:**
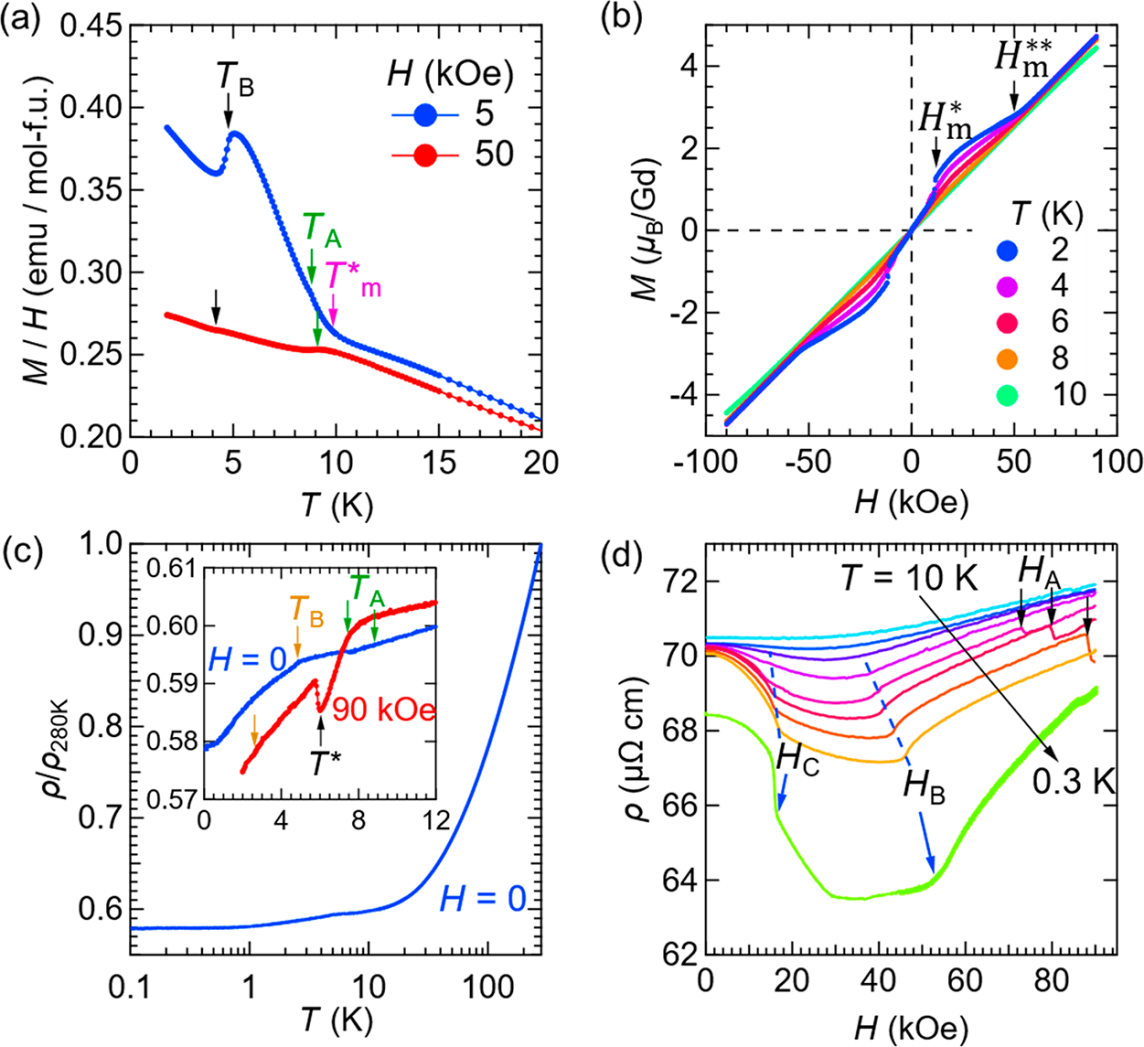
Magnetic and transport properties of GdAu_3_Si. (a) Temperature dependence of magnetization (plotted as *M*/*H*) under *H* = 5 kOe and 50 kOe at low temperatures. (b) *M*–*H* curves measured at several temperatures. The remarkable points, denoted by *T*_m_^*^, *H*_m_^*^, and *H*_m_^**^, are determined from the magnetization data. (c) Temperature dependence of the normalized electrical resistivity (ρ/ρ_280K_) under zero field; ρ_280K_ = 120 μΩ cm. The inset shows the close-up view of the low-temperature region including resistivity under the magnetic field of *H* = 90 kOe. (d) Magnetic-field dependence of electrical resistivity measured at *T* = 0.3, 5.5, 6.3, 7.2, 7.7, 8.0, 8.5, 9.0, and 10 K. The specific temperatures (*T*_A_, *T*_B_, and T*) and magnetic fields (*H*_A_, *H*_B_, and *H*_C_) determined by the specific heat data are indicated by the arrows and broken lines. We note that “mol-f.u.” in (a) refers to the formula unit GdAu_3_Si.

The GdAu_3_Si structure type provides a new type of magnetic sublattice forming a network of distorted icosahedron-like polyhedra (cf. Figure 5). We observe a Curie–Weiss behavior of the magnetic susceptibility *M*/*H* above ∼50 K with an effective magnetic moment of *p*_eff_ = 7.99 μ_B_/Gd (see Figure S10), which is in good agreement with the theoretical value for a free Gd^3+^ ion (7.94 μ_B_/Gd). The estimated Curie–Weiss temperature is θ_p_ ≈ −10 K, indicating that antiferromagnetic interactions are dominant. Figure 7a shows the temperature dependence of the magnetization (plotted as *M*/*H*) under the magnetic field of *H* = 5 and 50 kOe. We observe anomalies (denoted by *T*_A_, *T*_B_, and *T*_m_^*^) in the *M*–*T* curve. Figure 7b shows the *M*–*H* curves. We observe a slight meta-magnetic-like jump at *H*_m_^*^ ∼ 10 kOe and a linear behavior above *H*_m_^**^ ∼ 50 kOe, indicating possible changes in the configuration of magnetic order, yet the overall feature is of antiferromagnetic-type. See Figures S11–S15 for more detailed magnetization data. Figures 7c,d shows the temperature and magnetic field dependence of the electrical resistivity, respectively. In the ρ–*H* curve, we observe inflections at *H*_B_ and *H*_C_ for *T* < 8 K and steps at *H*_A_.

**Figure 8:**
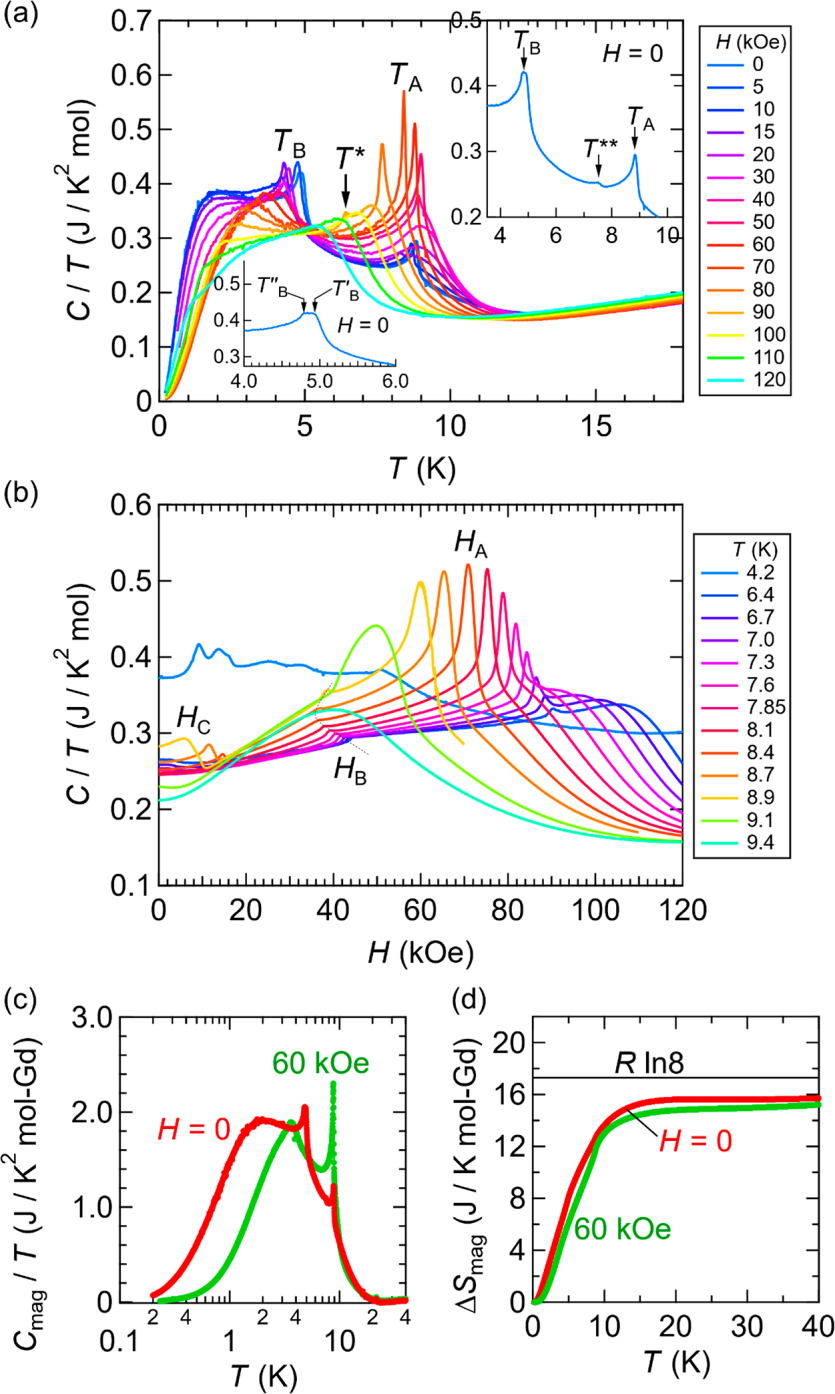
Specific heat of GdAu_3_Si. (a) Temperature dependence of specific heat divided by temperature (*C*/*T*) for various values of magnetic fields. The upper-right inset shows a close-up view for a small anomaly denoted by T**, while the lower-left inset shows a close-up view for the two inflections at *T*_B_ (denoted by *T*_B_^′^ and *T*_B_^′′^). Note that the two-inflection structure is absent for *H* ≥ 5 kOe. (b) Magnetic field dependence of *C*/*T*. (c) Magnetic contribution to the specific heat (*C*_mag_ /*T*). (d) Magnetic entropy measured from *T* ≈ 0.2 K (Δ*S*_mag_). Note that the unit “mol” in (a) and (b) indicates the mole of Gd_0.2_Au_0.6_Si_0.2_, while “mol-Gd” in (c) and (d) the mole of Gd atoms.

**Figure 9:**
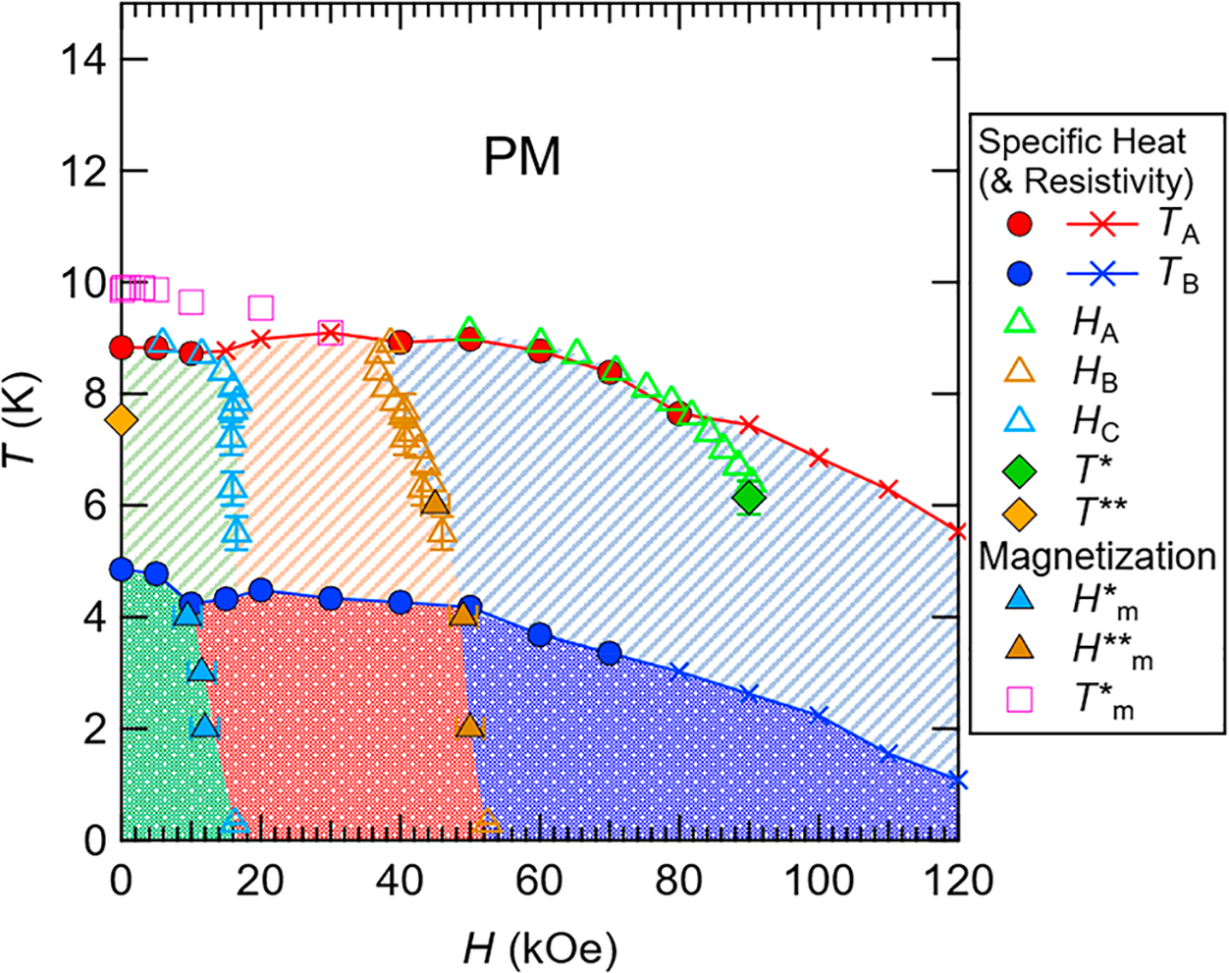
Characteristic data points (obtained from the specific heat, electrical resistivity and magnetization) plotted in the temperature vs magnetic field graph. For *T*_A_ and *T*_B_, the cross symbols indicate that the anomalies are broad peaks or inflections, while the circle filled symbols are from the sharp peaks. The acronym PM indicates a paramagnetic phase.

Figure 8a depicts the temperature dependence of the specific heat (plotted as *C*/*T*) under various magnetic fields at low temperatures. We observe two notable peaks corresponding to *T*_A_ and *T*_B_, suggesting two-step magnetic transitions. The peak at *T*_A_ is sharp for *H* ≤ 10 kOe, becomes broad in the range of 15 ≲ *H* ≲ 40 kOe, again becomes very sharp in 50 ≲ *H*≲ 80 kOe, and becomes broad above *H* ≳ 90 kOe (up to *H* = 120 kOe) with peak shifts toward lower temperatures as *H* increases. However, the peak at *T*_B_ shifts toward lower temperatures as *H* increases and turns into an inflection for *H* ≳ 80 kOe. The specific heat at *H* = 90 kOe exhibits an additional peak corresponding to T*. The zero-field specific heat also exhibits a small anomaly at T** and two inflections at *T*_B_ (denoted as *T*_B_^′^ = 4.94 K and *T*_B_^′′^ = 4.79 K) as shown in the insets of Figure 8a. Figure 8b depicts the magnetic field dependence of *C*/*T* at several temperatures around *T*_A_. We observed anomalies corresponding to *H*_A_, *H*_B_, and *H*_C_ in the resistivity measurements. We estimated the magnetic contribution to the specific heat (*C*_mag_) by estimating the lattice (phonon) contribution from the specific heat of YAu_3_Si (Figure 8c) and calculated the magnetic entropy (Δ*S*_mag_) above 0.2 K (Figure 8d). See the Supporting Information (and Figure S16 therein) for details. The magnetic entropy Δ*S*_mag_(*T*) seems to saturate near *R* ln 8 (where *R* is the gas constant) above the magnetic transition temperature *T*_A_, indicating that Gd^3+^ (*J* = 7/2) magnetic moments become free with almost full (2*J* + 1)-fold degeneracy (under crystal electric fields) above *T*_A_, which is in line with typical Gd compounds.^43^ Note that the deviation from the value of *R* ln8 may be attributed to the missing contribution from below the base temperature (0.2 K) and/or shortcomings in the estimated phonon contribution. Figure 9 shows the characteristic temperatures (*T*_A_, *T*_B_, T*, T**, and *T*_m_^*^) and magnetic fields (*H*_A_, *H*_B_, *H*_C_, *H*_m_^*^, and *H*_m_^**^) obtained from the specific heat, magnetization, and electrical resistivity. It seems there are three different magnetic states below *T*_A_, depending on the external magnetic field (as highlighted with green, red, and blue hatchings). Each state is further separated at *T*_B_. We observe a hysteresis behavior crossing the *H*_A_ line for 70 ≲ *H* ≲ 90 kOe, suggesting that the *H*_A_ line for 70 ≲ *H* ≲ 90 kOe (probably terminated near a specific point denoted by T*) is first-order-like.

**Figure 10:**
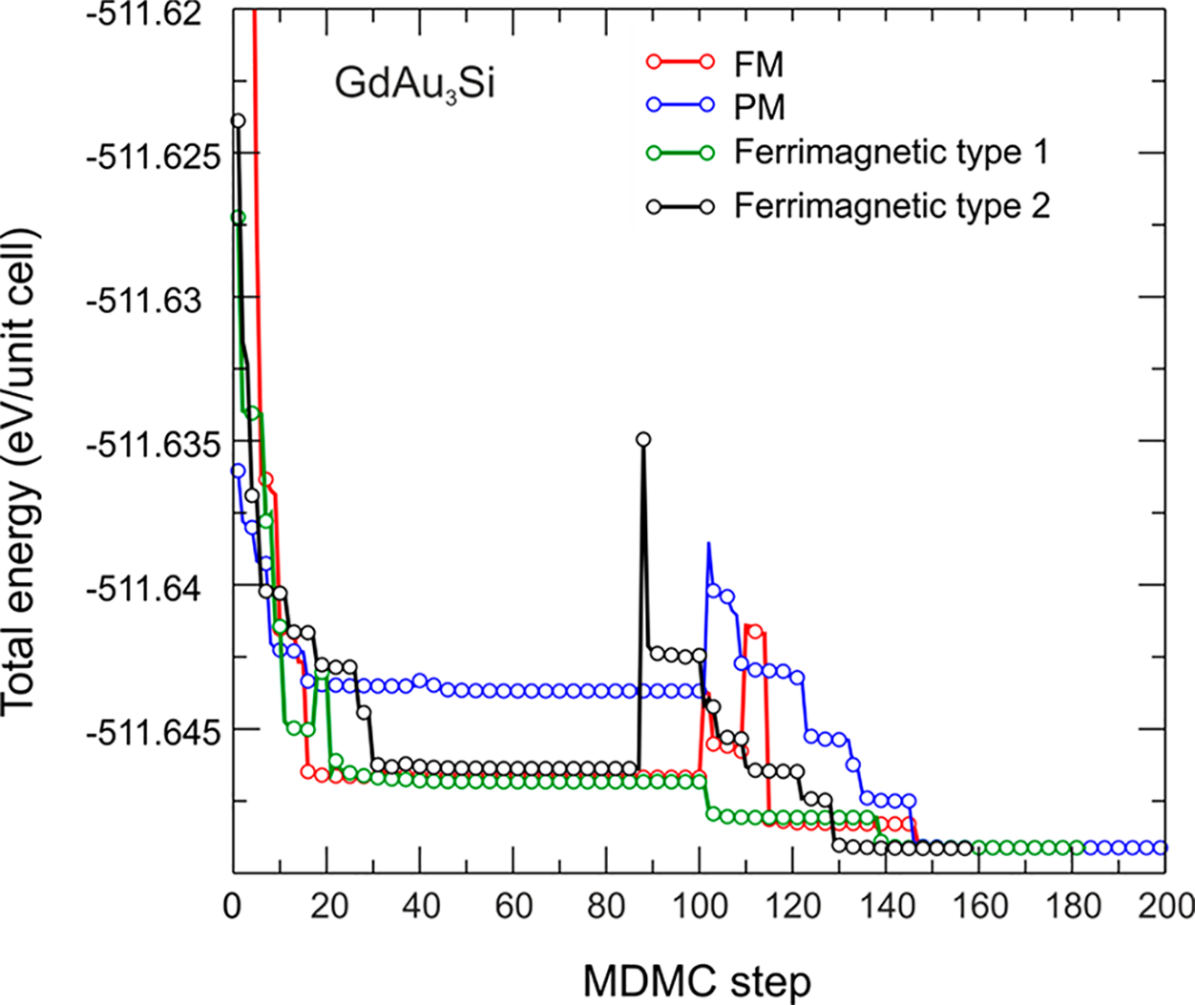
Relaxation of the total energy of GdAu_3_Si with four initial distributions of magnetic moments: FM (shown with red line), PM (shown with blue line), and two ferrimagnetic (shown with green and black lines). Each third step of the MDMC simulation is shown with spheres of the corresponding colors.

To shed more light into the complex magnetic behavior of GdAu_3_Si, MDMC simulations were carried out. For these simulations, four initial distributions of magnetic moments, namely, ferromagnetic (FM), paramagnetic (PM), and two types of the ferrimagnetic one, were considered (Figure 10). The paramagnetic distribution was created in a disordered local-moment (DLM) fashion^44^ known to nicely mimic the true paramagnetic distribution while keeping magnetic moments collinear and the total magnetic moment equal to zero. The ferrimagnetic distribution of type 1 was created with magnetic moments at atoms on the Gd1 (4*e*) and Gd3 (8*i*) positions parallel to each other, while magnetic moments on the Gd2 (4*f*) position were aligned antiparallel to them. The ferrimagnetic distribution of type 2 was created with magnetic moments at atoms on the Gd2 and Gd3 positions in parallel, while the moments on the Gd1 position were antiparallel to them. Several initial distributions were tested in order to prove the convergence of all the starting configurations to the same magnetic state with the lowest energy. In addition, two values of the starting magnetic moment (3 and 7 μ_B_ per atom) were tested which resulted always in a high-spin state with magnetic moments of Gd equal to 7.08–7.1 μ_B_. As already mentioned above, the resulting magnetic states were tested for a possible noncollinearity using the noncollinear version of VASP and appeared to be collinear.

**Figure 11:**
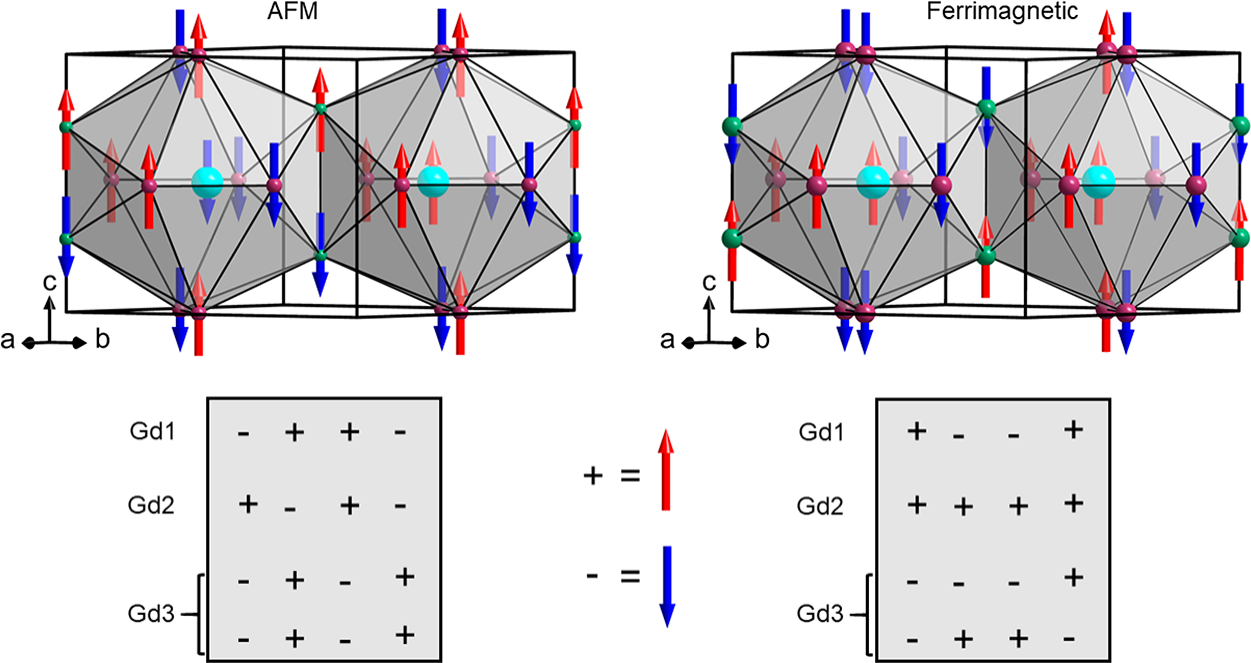
Distribution of magnetic moments in two neighboring icosahedral clusters Gd2(Gd1,Gd3)_12_ for the two magnetic states corresponding to lowest total energies, as identified from MDMC simulations.

As one can see from Figure 10, the initial FM distribution has the highest total energy, whereas the PM distribution has the lowest, which is rather close to the equilibrium magnetic state after the simulation. The energy differences between magnetic states are rather small. After approximately 160 MDMC steps, all considered initial distributions of magnetic moments converged to two configurations, which were very close in energy and remained unchanged through further MDMC runs. These two final states are antiferromagnetic (AFM), with 8 parallel and 8 antiparallel spins, and ferrimagnetic, with 9 parallel and 7 antiparallel spins (for the particular distributions, see Figure 11). Both states have collinear magnetism. As they are very close in energies (which should be considered as zero within the DFT accuracy), one might expect a frustrated magnetic behavior of the system. The collinear magnetic order suggests the presence of an easy-axis anisotropy.

## 4 Discussion

The complex *T*–*H* phase diagram shown in Figure 9, which includes several regions of *T*–*H* in which the magnetic order is modulated, is reminiscent of centrosymmetric frustrated antiferromagnets (Gd-based intermetallic compounds) such as triangular lattice Gd_2_PdSi_3_^45^ and breathing-kagome-lattice Gd_3_Ru_4_Al_12_^46^ whose intriguing chiral (topological) spin textures called skyrmion lattices were recently reported. Similar to these Gd-based frustrated antiferromagnets, GdAu_3_Si has a centrosymmetric crystal structure (spatial inversion symmetry) with triangular magnetic units (which could cause geometrical frustration). Qualitatively one can assume the same exchange-coupling mechanism, i.e., Ruderman–Kittel–Kasuya–Yosida (RKKY)-type exchange interaction, which is also plausible from the DOS of GdAu_3_Si (cf. Figure S5b). However, GdAu_3_Si has a three-dimensional crystal structure (rather than a (quasi) two-dimensional), and the triangular units constituting the icosahedra are distorted (cf. Figure 5b). The quasi-degenerate feature (energetically close-lying magnetic states) found in our simulation and the complex magnetic behavior for *H* = 0, which is represented by additional anomalies in the specific heat (i.e., the anomaly at T** and the two-inflection behavior at *T*_B_), may reflect magnetic frustration. The complex *T*–*H* phase diagram of GdAu_3_Si also bears some resemblance to those of noncentrosymmetric chiral-lattice magnets (with broken inversion symmetry) such as metallic MnSi^47^ and insulating (multiferroic) Cu_2_OSeO_3_.^48^ We note that the inversion symmetry with respect to magnetic Gd atoms is locally broken since the Gd2(Gd1,Gd3)_12_ pseudo-icosahedron with the cluster-center Gd2 (see Figure 5b) does not have inversion symmetry, though the global inversion symmetry is conserved.

We also analyzed the critical behavior of the specific heat at the magnetic transition at *T*_A_ for *H* = 0, 5 and 60 kOe considering the minimal curve *C* = *A*_±_|*t*|^–α^ + *B* + *Lt*,^49^ in which the first term describes a critical behavior, while the last two terms represent background contributions, where α, *A*_*±*_, B, L are adjustable parameters and *t* ≡ (*T* – *T*_N_)/*T*_N_ (with *T*_N_ ≈ *T*_A_); see the Supporting Information (and Figure S17 therein) for details. α is a critical exponent, and the subscript “+” and “–” indicates *T* > *T*_N_ and *T* < *T*_N_, respectively. Our analysis suggests an approximate critical exponent of α = 0.2 ± 0.04 with *A*_+_ /*A*_–_ ∼ 0.66 for *H* = 0 and 5 kOe (the green region in the *T*–*H* diagram (see Figure 9), and α = 0.13 ± 0.03 with *A*_+_/*A*_–_ ∼ 0.82 for *H* = 60 kOe (the blue region). The latter α value is close to those of the universality class of 3D-Ising (α = 0.11, *A*_+_ /*A*_–_ = 0.52).^49^ This is in line with our computational result suggesting a uniaxial anisotropy. The low-field analysis (*H* = 0 and 5 kOe) yields parameters closer to those of the “chiral” Heisenberg type (α = 0.24 ± 0.08, *A*_+_/*A*_–_ = 0.54 ± 0.2).^50^

Interestingly, there is a qualitative similarity between the present GdAu_3_Si system and the quasi-two-dimensional triangular Heisenberg frustrated antiferromagnets referred to as VX_2_ (X = Cl, Br, and I), which have been studied with respect to the “chiral” universality class.^50,51^ This similarity between these completely different systems (having different structures and exchange-coupling mechanisms) may be due to the universality of the “chiral” Heisenberg class.^50^ The VX_2_ systems exhibit sharp peaks in their specific-heat curves at their magnetic transitions. According to refs (50) and (52), VCl_2_ has a weak Ising-like anisotropy which causes two successive transitions in a very narrow temperature range, which is similar to the two-inflection structure at *T*_B_ observed in the specific heat (*H* = 0) of GdAu_3_Si. We therefore conjecture that the uniaxial anisotropy (with the chiral Heisenberg behavior) plays an important role in GdAu_3_Si. Also, VI_2_ exhibits two distinct successive transitions (with respect to a transition to a 120° spin structure and to a collinear spin structure).^53^ The latter exhibits a very sharp peak in its specific heat, which is similar to the very sharp peaks observed in the present system at *T*_A_ in 50 ≲ *H* ≲ 80 kOe. From the similarity to VX_2_ and the estimated critical exponents, it appears reasonable to suggest that GdAu_3_Si has a chiral–Heisenberg nature (with a uniaxial anisotropy) in the low-field region (the green region, *H* ≲ 50 kOe) and exhibits an Ising-like nature at the high-field region (the blue region, *H* ≲ 50 kOe).

## 5 Conclusions

The investigation of reaction mixtures RE_*x*_(Au_0.79_Si_0.21_)_100–*x*_ (RE = Y and Gd) resulted in the compounds YAu_3_Si and GdAu_3_Si which crystallize in a new tetragonal structure type. The strictly (Au, Si) ordered structure features three crystallographically different RE atoms which are each coordinated by 12 Au and 4 Si atoms. The RE(Au,Si)_16_ polyhedra form interpenetrating frameworks in which the RE atom substructure corresponds to a bcc-like arrangement of centered icosahedra. Nonmagnetic YAu_3_Si exhibits conventional BCS type-II superconductivity around 1 K. Antiferromagnetic GdAu_3_Si exhibits a multifarious *T*–*H* phase diagram, which reflects its complex low temperature (*T* ∼ 10 K) magnetic order. To characterize the *T*–*H* phase diagram suggested in Figure 9, further investigations are required. Unfortunately, neutron-scattering experiments are hampered by the extraordinarily high absorption cross section of Gd. Similar to the compositionally neighboring cubic 1/1 AC phases (RE(Au,Si)_∼6_), REAu_3_Si phases may be afforded for a larger range of RE which would give the opportunity for broader physical property studies associated with RE magnetism.

## Supporting Information Available

Plots of SEM/EDX, PXRD, DOS, and electron density iso-surface for YAu_3_Si; list of interatomic distances for REAu_3_Si (RE = Gd and Y); plots of electrical resistivity and specific heat of YAu_3_Si; plots of magnetization and specific heat of GdAu_3_Si (PDF)

## Accession Codes

CCDC 2117650 and 2117652 contain the supplementary crystallographic data for this paper. These data can be obtained free of charge via www.ccdc.cam.ac.uk/data_request/cif, or by emailing data_request@ccdc.cam.ac.uk, or by contacting The Cambridge Crystallographic Data Centre, 12 Union Road, Cambridge CB2 1EZ, UK; fax: +44 1223 336033.
