# Supplementary material for: Superconducting YAu3Si and Antiferromagnetic GdAu3Si with an Interpenetrating Framework Structure Built from 16-Atom Polyhedra
Source: Inorg Chem. 2022 Feb 28;61(10):4322–34. doi: 10.1021/acs.inorgchem.1c03456 (PMC8924926; doi:10.1021/acs.inorgchem.1c03456)
Supplement: Supplementary file 1 — ic1c03456_si_001.pdf [file ic1c03456_si_001.pdf]

Supporting Information for

Superconducting YAu_3Si and antiferromagnetic $GdAu_3Si$ with an interpenetrating framework structure built from 16 atom polyhedra.

Girma Hailu Gebresenbut,[1,] Lars Eriksson,[2] Ulrich Häussermann,[2] Andreas Rydh,[3] Roland Mathieu,[4] Olga Vekilova,[2] Takayuki Shiino [4,*]*

[1]Department of Chemistry-Ångström Laboratory, Uppsala University, 751 21 Uppsala, Sweden

[2]Department of Materials and Environmental chemistry, Stockholm University, 106 91 Stockholm, Sweden

[3]Department of Physics, Stockholm University, 106 91 Stockholm, Sweden

[4]Department of Materials Science and Engineering, Uppsala University, Box 35, 751 03 Uppsala, Sweden

* girma.gebresenbut@kemi.uu.se

* takayuki.shiino@angstrom.uu.se

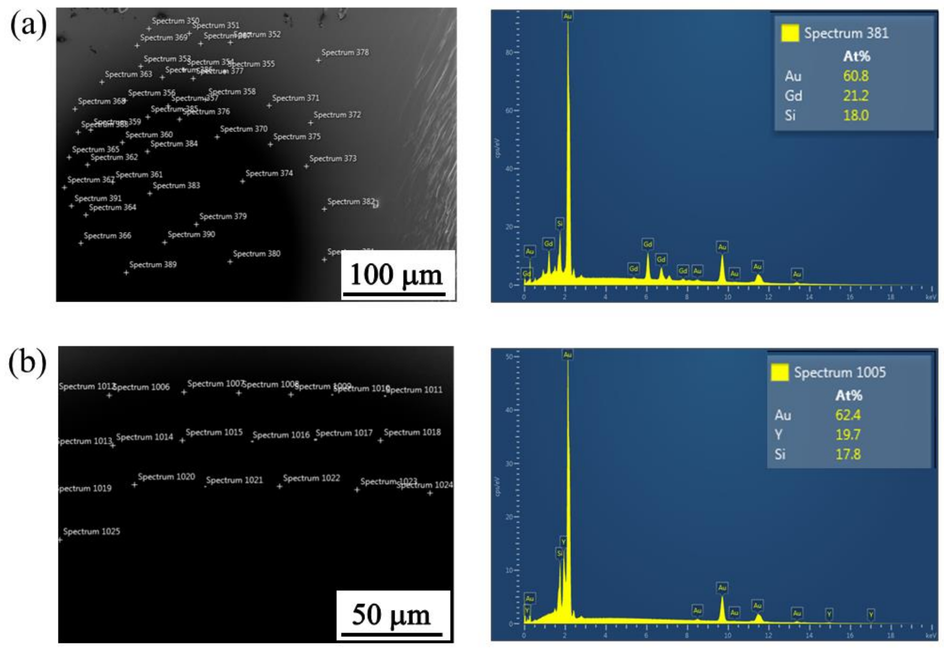

Fig. S1. Backscattered SEM images (left) of cross-section polished samples and representative EDX spectra of analyzed regions (right) for (a) $GdAu_3Si$ and (b) YAu_3Si.

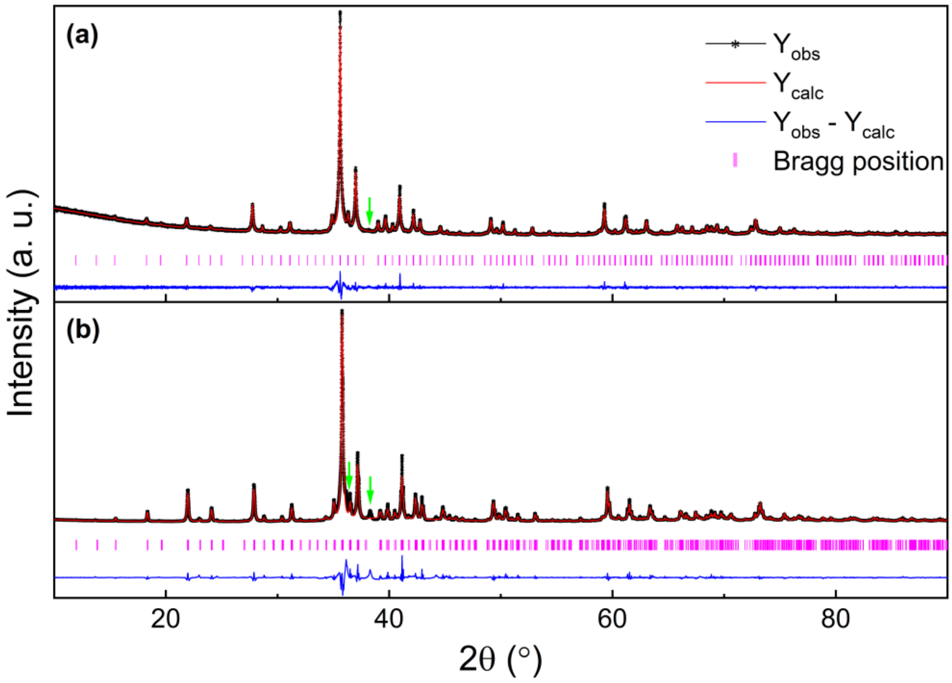

Fig. S2. Whole-pattern PXRD profile fitting for (a) $GdAu_3Si$ (χ^2 = 1.21, R_B = 3.11, R_F = 1.96) and (b) YAu_3Si (χ^2 = 20.80, R_B = 5.75, R_F = 3.44) using the FullProf Suite. Crystal structure models obtained from SCXRD refinements of the respective compounds were used as a model. Diffraction peaks from residual Au-Si flux is indicated by green arrows. Note that the pattern in (b) contains Cu-$K_{\alpha2}$ radiations and an attempt to remove it resulted to unstable fit.

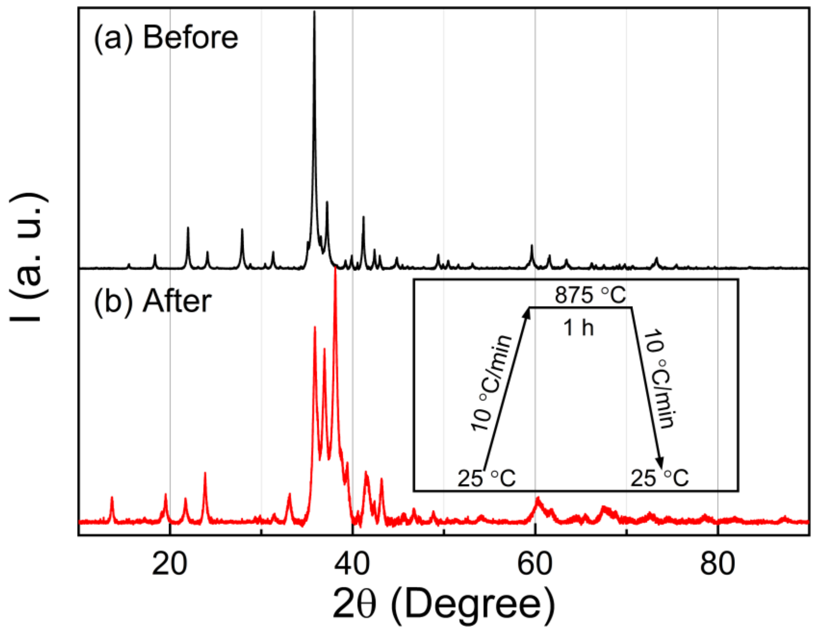

Fig. S3. PXRD patterns for YAu_3Si (a) before and (b) after annealing for 1 h at 875 °C. X-ray scattering background and diffraction peaks from Cu-$K_{\alpha 2}$ radiation have been removed from each pattern for clarity. The inset diagram shows the annealing protocol used in the annealing experiment. The PXRD patterns before and after the DSC experiment are different indicating the YAu_3Si phase is thermally unstable.

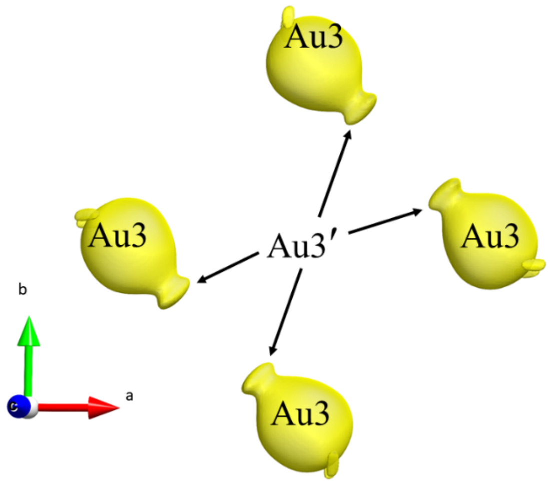

Fig. S4. Electron density iso-surfaces near the Au3/Au3′ atomic position for YAu_3Si. The observed positional disorder was modeled as two mutually exclusive (split) Au3 and Au3′ sites; the sum of their site occupancy factors (S.O.F.) were constrained to unity. Iso-surfaces are obtained by standard Fourier maps using the observed SCXRD intensities. The iso-surface level is set at 15 eÅ^{-3}.

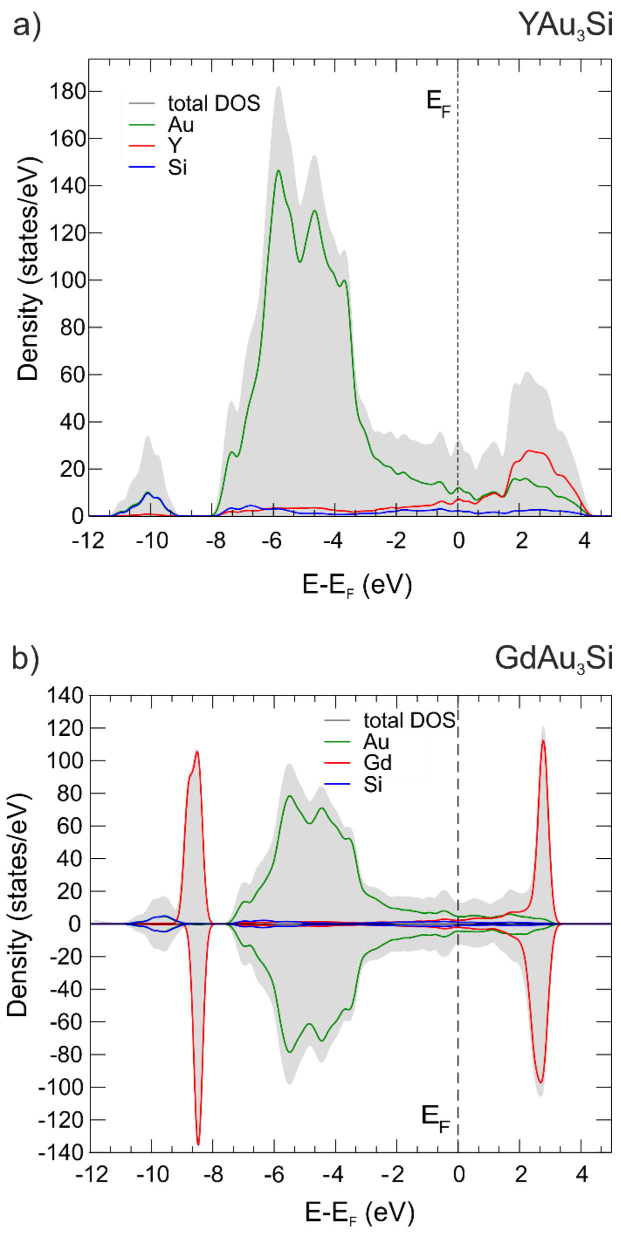

Fig. S5. Electronic density of states of YAu_3Si (a) and $GdAu_3Si$ (b). The latter corresponds to the lowest energy AFM structure as established from MDMC simulations. The total DOS is represented by the grey area whereas partial atomic contributions are depicted by the colored lines (Au-green, RE-red, Si-blue). Note that the Fermi level of YAu_3Si is located at a maximum of the DOS, which could relate to the superconducting ground state.

Table S1. Interatomic distances for $REAu_3Si$ (RE = Gd, Y) obtained from SCXRD refinements.

$GdAu_3Si$				YAu_3Si				YAu_3Si			
**Atom pair**			**d/Å (< 3.5 Å)**	**Atom pair**			**d/Å (< 3.5 Å)**	**Atom pair**			**d/Å (< 3.5 Å)**
Gd1	Au4	2x	3.1266(7)	Y1	Au3'	4x	3.08(1)	Au3'	Au3	1x	0.42(2)
	Si1	4x	3.175(4)		Au2	2x	3.1120(7)		Au3'	1x	1.91(2)
	Au1	4x	3.218(1)		Si1	4x	3.168(3)		Si1	2x	2.619(9)
	Au3	4x	3.230(1)		Au1	4x	3.200(2)		Au3'	1x	2.83(2)
	Au2	2x	3.2765(6)		Au3	4x	3.227(3)		Au4	2x	2.88(1)
Gd2	Au3	2x	3.031(1)		Au4	2x	3.2664(7)		Au3'	1x	3.41(2)
	Au2	2x	3.084(1)	Y2	Au3	2x	2.998(3)	Au4	Si1	2x	2.573(3)
	Au5	4x	3.191(1)		Au4	2x	3.062(2)		Au5	2x	3.2148(8)
	Au1	2x	3.239(1)		Au5	4x	3.178(2)	Au5	Si1	1x	2.506(3)
	Au4	2x	3.252(1)		Au1	2x	3.221(2)		Si1	1x	2.543(3)
	Si1	4x	3.445(4)		Au2	2x	3.2385(9)		Au5	1x	2.8117(8)
Gd3	Si1	2x	3.117(4)		Au3'	2x	3.38(2)		Au5	2x	2.9882(7)
	Au3	1x	3.126 (1)		Si1	4x	3.446(3)		Au5	1x	3.4646(7)
	Au4	2x	3.130(1)	Y3	Si1	2x	3.104(3)				
	Au5	2x	3.1733(6)		Au3	1x	3.111(3)				
	Au5	2x	3.179 (1)		Au2	2x	3.118(1)	**RE – RE (< 6 Å)**			
	Au5	2x	3.185 (1)		Au5	2x	3.1591(6)	Gd1	Gd1	1x	3.942(2)
	Au1	1x	3.270 (1)		Au5	2x	3.164(1)		Gd2	2x	4.782(1)
	Au2	2x	3.276 (1)		Au5	2x	3.169(1)		Gd1	1x	5.146(2)
	Si1	2x	3.377(4)		Au3'	1x	3.24(2)		Gd2	2x	5.409(1)
Au1	Si1	2x	2.592(5)		Au1	1x	3.255(2)		Gd3	4x	5.561(1)
	Au1	1x	2.812(1)		Au4	2x	3.261(1)		Gd3	4x	5.581(1)
	Au5	2x	3.038(1)		Si1	2x	3.351(3)	Gd2	Gd3	2x	4.731(2)
	Au4	2x	3.058 (1)	Au1	Si1	2x	2.570(3)		Gd3	4x	5.1251(8)
	Au2	2x	3.0774(9)		Au1	1x	2.8014(9)		Gd3	2x	5.425(2)
Au2	Si1	2x	2.584(4)		Au5	2x	3.0315(8)	Gd3	Gd3	1x	4.514(2)
	Au4	1x	2.8214(9)		Au2	2x	3.0409(8)		Gd3	1x	4.563(2)
	Au3	2x	3.0513(9)		Au4	2x	3.0678(8)		Gd3	4x	5.5530(9)
	Au5	2x	3.2263(9)		Y1	2x	3.200(2)				
Au3	Si1	2x	2.439(4)		Y2	1x	3.221(2)	Y1	Y1	1x	3.922(4)
	Au3	1x	2.697(1)		Y3	1x	3.255(2)		Y2	2x	4.771(2)
	Au3	1x	2.824 (1)	Au2	Si1	2x	2.443(3)		Y1	1x	5.123(4)
Au4	Si1	2x	2.452(4)		Au4	1x	2.8061(7)		Y2	2x	5.376(2)
	Au4	1x	2.868(1)		Au2	1x	2.859(1)		Y3	4x	5.536(2)
Au5	Si1	1x	2.518(4)	Au3	Au3'	1x	0.42(2)		Y3	4x	5.559(2)
	Si1	1x	2.564(4)		Au3'	1x	2.32(2)	Y2	Y3	2x	4.716(2)
	Au5	1x	2.8256(8)		Si1	2x	2.430(3)		Y3	4x	5.100(1)
	Au5	2x	3.0049(9)		Au3	1x	2.747(4)		Y3	2x	5.395(2)
	Au5	1x	3.4859(9)		Au3	1x	2.801(4)	Y3	Y3	1x	4.493(2)
					Au3'	1x	2.85(2)		Y3	1x	4.545(2)
					Au4	2x	3.057(2)		Y3	4x	5.527(1)

Supplementary data for the electrical resistivity of YAu_3Si

Figure S6 shows the magnetic-field dependence of the electrical resistivity of YAu_3Si. We plot the upper critical field (H_{c2}) vs temperature in the inset of Figure 6(b) in the main text using this data.

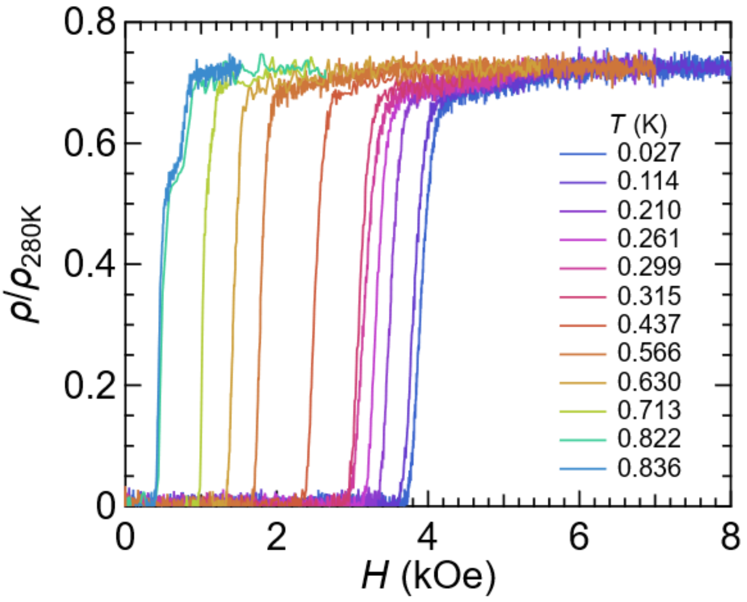

Fig. S6. Magnetic-field dependence of the electrical resistivity of YAu_3Si measured at several temperatures.

In this study, we have noticed that a small amount of Y-Au-Si approximant crystal referred to as YAS(CC)[1] is included in our YAu_3Si samples. Figures S7(a) and (b) show the resistivity data of another YAu_3Si sample (named "Sample #2") for comparison. We observe double-step behaviors at the superconducting transition in the ρ-T and ρ-H curves. We plot the first-drop temperature (T_c') and magnetic field (H_{c2}') in Figure S7(c). The H_{c2}' vs T curve is almost the same as that of YAS(CC), indicating that the double-step features (i.e., the first drops) observed in our YAu_3Si samples are due to slightly included YAS(CC) impurity phase, which cannot be detected in the powder x-ray measurements. Note that Sample #2 was synthesized in a slightly different condition from that for our main batch sample presented in the main text as follows: the starting composition is $Y_{14}(Au_{0.79}Si_{0.21})_{86}$ and after the melting, it was slowly cooled down from 910 ℃ to 830 ℃ for crystallization.

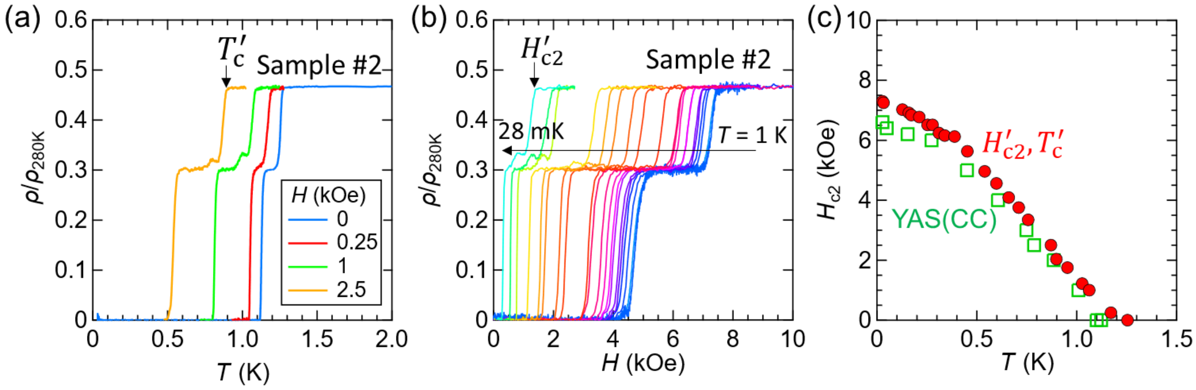

Fig. S7. Superconductivity of YAu_3Si (Sample #2). (a) Temperature dependence of the electrical resistivity (plotted as ρ/ρ_{280K} where ρ_{280K} is the value at 280 K) under several magnetic fields. (b) Magnetic-field dependence of the electrical resistivity measured at different temperatures. (c) The H_{c2}' vs T curve plotted with the H_{c2} vs T data of YAS(CC).

Supplementary information for the specific heat of YAu_3Si

Figure S8(a) shows the raw specific heat data of YAu_3Si measured under various magnetic fields near the superconducting transition temperature. The peak temperature points are plotted in the inset of Figure 6(b) in the main text. The broken curve indicates an estimated background specific-heat data contributed from the calorimeter at low temperatures, which exhibits an upturn behavior and a magnetic-field dependence. Note that this background contribution is estimated based on similar calorimeters used in our previous studies. This makes it difficult for us to subtract the background contribution from the specific heat under magnetic fields. For the zero-field specific heat data, we subtract this background.

Figure S8(b) shows the C/T vs T^2 plot for the zero-field specific-heat data. From the linear fitting above T_c, we obtain the specific-heat coefficients $\gamma_n = 1.1$ mJ/K^2 mol and $\beta = 0.10$ mJ/K^4 mol (i.e., $C/T = \gamma_n + \beta T^2$).

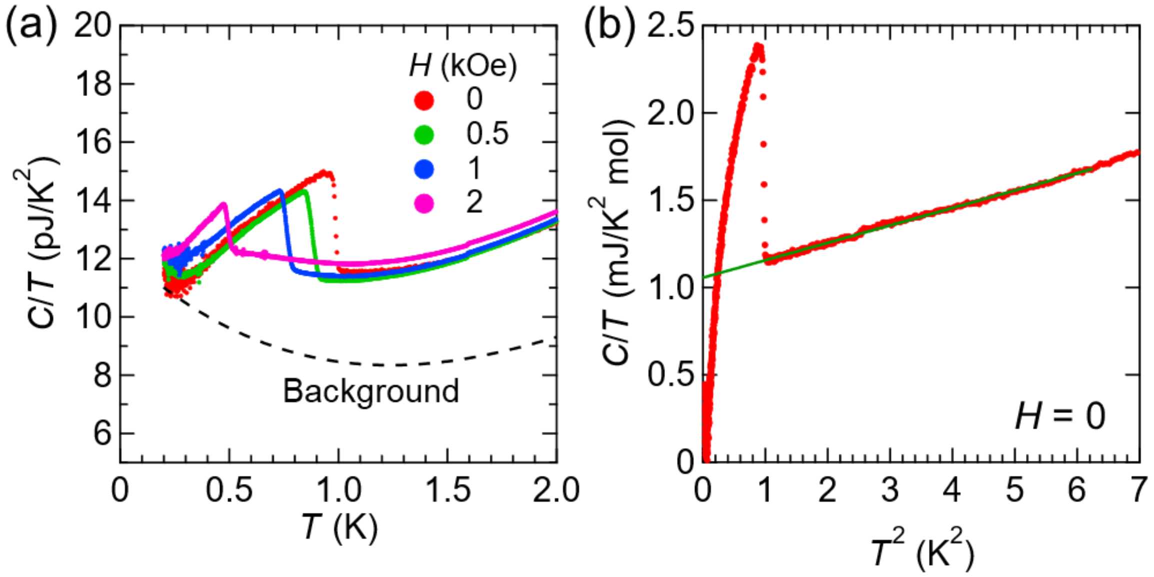

Fig. S8. Specific heat of YAu_3Si. (a) Raw specific heat data for various magnetic fields. The broken curve indicates an estimated background of a calorimeter for zero fields ($H = 0$). Note that this background calorimeter contribution changes under magnetic fields. (b) C/T vs T^2 plot for the zero-field data. The green line indicates the linear fit from the normal phase. Note that 'mol' in (b) indicates the mole of $Y_{0.2}Au_{0.6}Si_{0.2}$.

Estimation of the thermodynamic critical field for YAu_3Si

We estimate the thermodynamic critical field H_c using the following relation:

$$-\frac{\mu_0 H_c(T)^2}{2} = \Delta F(T) = \Delta U(T) - T\Delta S(T),$$

where

$$\Delta U(T) = \int_{T_c}^{T} \Delta C(T')dT',$$
$$\Delta S(T) = \int_{T_c}^{T} \frac{\Delta C(T')}{T'} dT'.$$

This equation indicates superconducting condensation energy. Note that $\Delta C(T) = C_s(T) - C_n(T)$, where C_s is the specific heat in the superconducting state, while C_n is that in the normal state. Note that $\Delta U(T_c) = \Delta S(T_c) = 0$. We plot the calculation results in Figure S9: (a) $\Delta U, T\Delta S$ and ΔF, (b) the thermodynamic critical field H_c. We compare the estimated $H_c(T)$ with the following model curve:

$$H_c(T) \approx H_c(0)\left[1 - \left(\frac{T}{T_c}\right)^2\right]$$

where we set $T_c = 0.97$ K. The absolute-zero value $H_c(0)$ was estimated from extrapolation using the following polynomial fitting equation: $H_c(T) = c_1 + c_2T^2 + c_3T^4$ where c_1, c_2 and c_3 are fitting parameters. The model curve and fitting result are plotted in Figure S9(b). The negative deviations from the model in the intermediate temperature region indicate weak-coupling behavior[2].

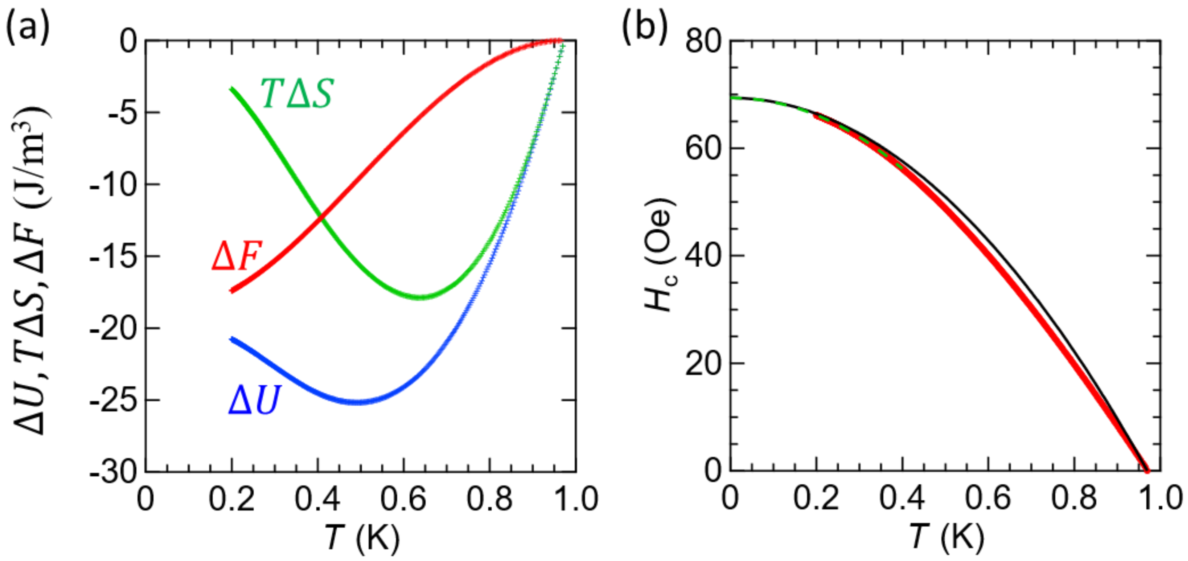

Fig. S9. (a) Differences of internal energy (ΔU), entropy (plotted as $T\Delta S$), and free energy (ΔF). (b) Thermodynamic critical field H_c. The green broken curve indicates an extrapolation to $T = 0$ from the fitting (see the text), while the black solid curve indicates the model curve.

Supplementary information for the magnetization of $GdAu_3Si$

Curie-Weiss behavior of $GdAu_3Si$

Figure S10 shows the temperature dependence of the inverse magnetic susceptibility of the 4f-electron component $1/\chi_{4f}$. Note that $\chi_{4f} = M/H - \chi_0$ where M is magnetization and χ_0 is the temperature-independent non-4f-electron contribution, whose value was determined so that the curve exhibits a linear temperature dependence. This indicates that χ_{4f} exhibits Curie-Weiss law, i.e., $\chi_{4f} \propto p_{\rm eff}^2/(T-\theta_{\rm p})$. The value of effective magnetic moment extracted from the experimental data ($p_{\rm eff} = 7.99\ \mu_{\rm B}$/Gd) is in good agreement with the theoretical value of a free Gd^{3+} ion ($g_J\sqrt{J(J+1)} = 7.94\ \mu_{\rm B}$/Gd) where g_J is Lande's g-factor. The Curie-Weiss temperature is $\theta_{\rm p} \approx -10$ K, indicating that antiferromagnetic interactions are dominant. The value of $\theta_{\rm p} \approx -10$ K is reasonable compared to the magnetic order temperature $T_{\rm A} \sim 9$ K.

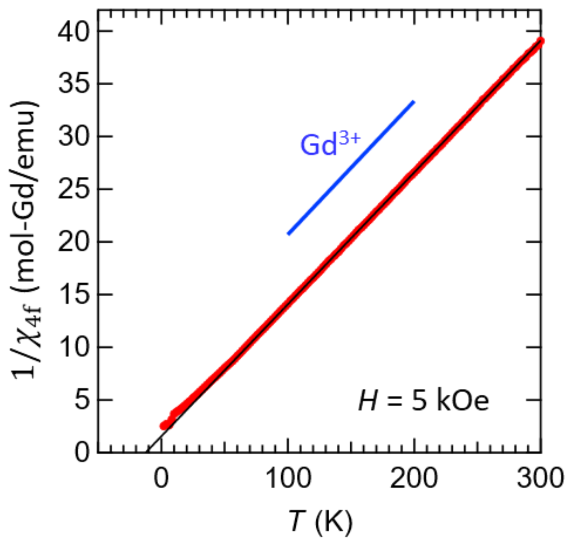

Fig. S10. Temperature dependence of the inverse magnetic susceptibility ($1/\chi_{4f}$) of 4f-electron contribution. The blue line indicates the slope for the free moment of Gd^{3+}.

Low-temperature magnetization of $GdAu_3Si$

Figure S11(a) shows the temperature dependence of the magnetization of $GdAu_3Si$ under various external magnetic fields. The anomaly at ~16 K observed for the low-field data can be attributed to the ferromagnetic transition of a minor amount of an impurity phase of Gd-Au-Si 1/1 quasicrystal approximant referred to as GAS(CC)[3]. Figure S11(b) shows the sample dependence of the magnetization under $H = 100$ Oe. Note that we do not observe any anomaly at ~16 K in the specific heat. The anomaly at ~16 K differs depending on the samples. We used sample-#1 as a main sample for the magnetic property measurements because sample-#1 seems to contain less impurity phase.

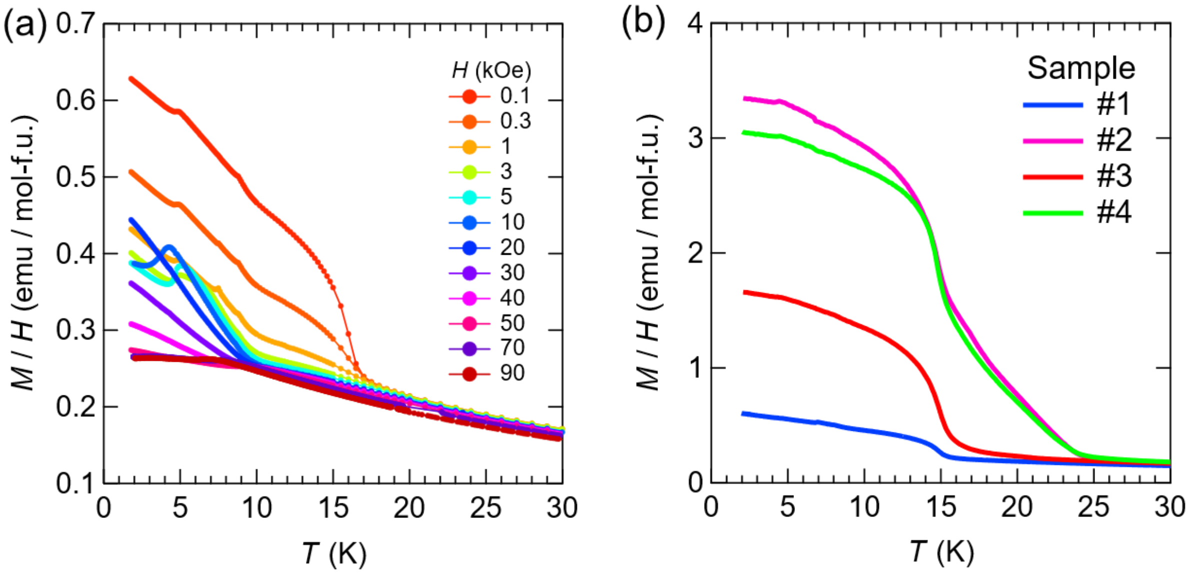

Fig. S11. (a) Temperature dependence of the magnetization (plotted as M/H) of $GdAu_3Si$ under various external magnetic fields. (b) M/H vs T curves (measured under $H = 100$ Oe) for several samples (#1-#4). Note that sample-#1 is the main sample used in this study. Note that 'mol-f.u.' refers to the formula unit $GdAu_3Si$.

Figure S12 shows the M/H vs T curves plotted separately for each H value. We observe anomalies in the M-T curves, which correspond to T_{A}, T_{B} T^{*}, and T^{**}. The M-T curve also seems to behave differently depending on the applied magnetic field, characterizing the three magnetic states shown in the T-H diagram (see Figure 9 in the main text). We do not clearly observe an anomaly at T_{A} for $3 \leq H \leq 20$ kOe (in the green region), and we do not at T_{B} for $20 \leq H \leq 40$ kOe (in the red region). For $H \lesssim 20$ kOe, we observe an inflection in the magnetization curve at $T_{\mathrm{m}}^{*} > T_{\mathrm{A}}$.

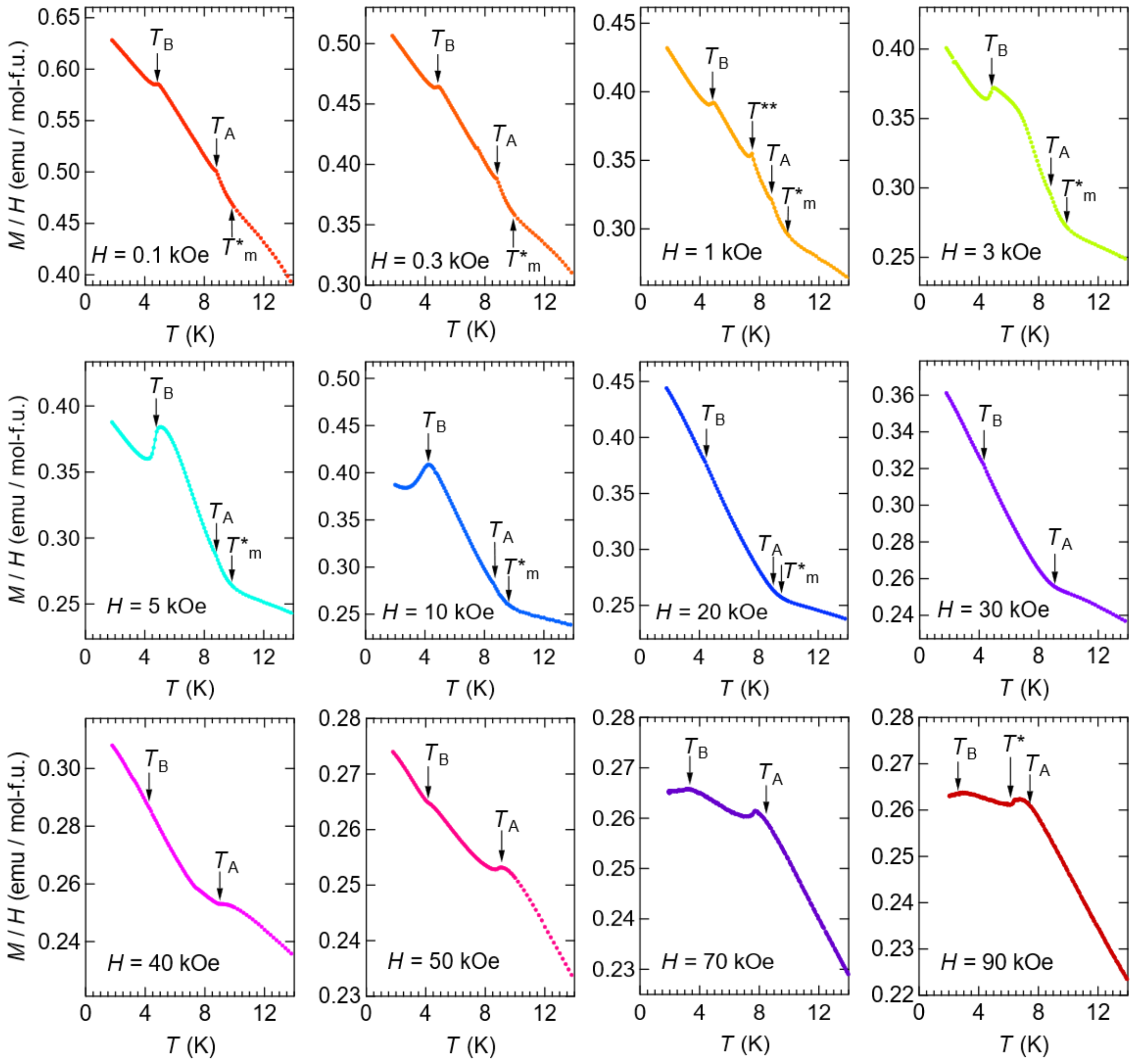

Fig. S12. Temperature dependence of the magnetization (plotted as M/H) of $GdAu_3Si$ under various magnetic fields at low temperatures. The characteristic temperatures, T_{A}, T_{B}, T^{*} and T^{**}, are indicated. The temperature denoted as T_{m}^{*} indicates the inflection of the magnetization curve at $T > T_{\mathrm{A}}$ for $H \leq 20$ kOe. Note that 'mol-f.u.' refers to the formula unit $GdAu_3Si$.

Hysteresis behaviors in $GdAu_3Si$

Figure S13 shows the temperature dependence of the magnetization of $GdAu_3Si$ around the transition temperature T_A under magnetic fields of $H \gtrsim 50$ kOe. For $H \gtrsim 70$ kOe, we observe a hysteresis behavior at the transition (crossing the H_A line shown in Figure 9 in the main text), which becomes significant as the magnetic field increases. Figure S14 shows the magnetic field dependence of the magnetization. Hysteresis behaviors are also observed in the *M*-*H* curve at $(H, T) = (81.5 \text{ kOe}, 6.5 \text{ K})$ and $(76\text{kOe}, 7 \text{ K})$. These points are near the H_A line. We do not observe a clear anomaly for $T \geq 7.5$ K. These hysteresis behaviors suggest that the H_A line for $70 \lesssim H \lesssim 90$ kOe is first-order-like.

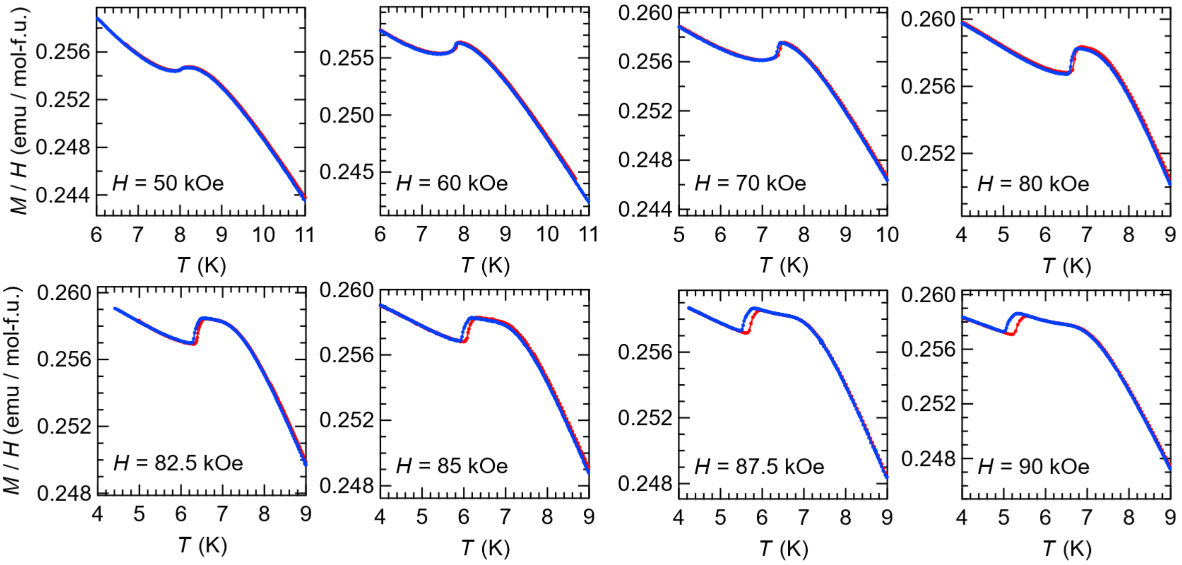

Fig. S13. Magnetic-field dependence of the magnetization (plotted as M/H) of $GdAu_3Si$ at low temperatures. The data were collected in the cooling (blue) and warming (red) processes. Note that 'mol-f.u.' refers to the formula unit $GdAu_3Si$.

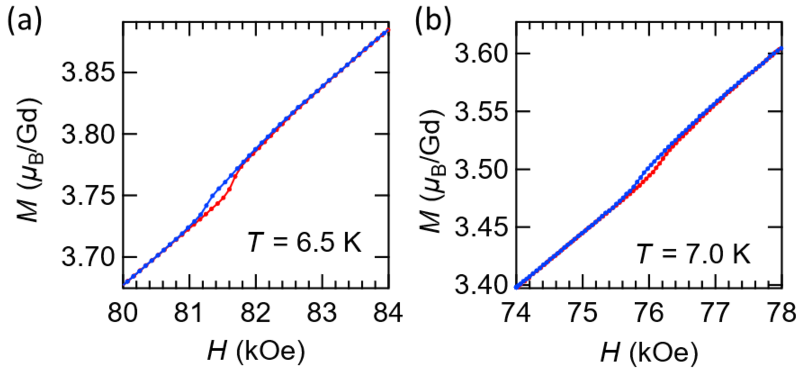

Fig. S14. Magnetic field dependence of the magnetization at the temperatures (a) $T = 6.5$ K and (b) $T = 7$ K. The data were collected in the decreasing (blue) and increasing (red) processes of the magnetic field.

Supplemental data for *M* vs *H* curves of $GdAu_3Si$

Figure S15 shows the *M*-*H* curve of $GdAu_3Si$ measured in detail using the SQUID magnetometer for the low-field region. We observe a meta-magnetic-like jump at H^*_{m} for the temperatures below T_{B} (i.e., $T = 2, 3$ and 4 K) and deviations from the linear behavior at the H_{C} line (i.e., $T = 5, 6, 7$ and 8 K). See also Figure 9 in the main text.

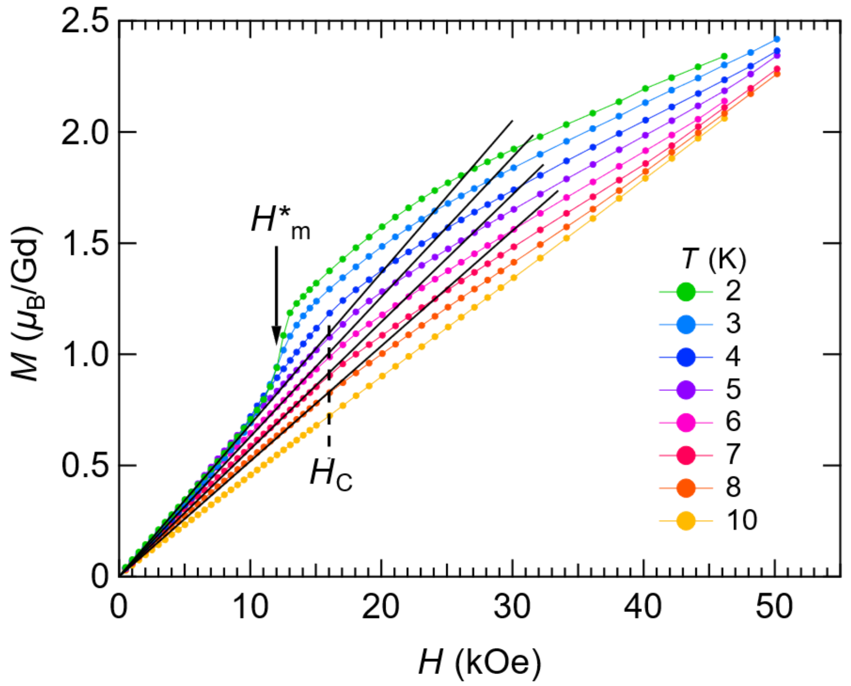

Fig. S15. *M*-*H* curve measured using the SQUID magnetometer for the low-field region up to 50 kOe. The black solid lines are guides for eyes for the linear behaviors at the low-field region for the $T = 5, 6, 7$ and 8 K data.

Supplementary information for specific heat of $GdAu_3Si$

Estimation of the magnetic contribution to the specific heat of $GdAu_3Si$

To estimate the lattice (phonon) contribution of $GdAu_3Si$, we use a scaling factor of $\theta_{\mathrm{GdAu_3Si}}/\theta_{\mathrm{YAu_3Si}} = 0.89$ where $\theta_{\mathrm{GdAu_3Si}} = 170$ K and $\theta_{\mathrm{YAu_3Si}} = 190$ K is a roughly estimated Debye temperature of $GdAu_3Si$ and YAu_3Si, respectively. We estimate the phonon contribution of $GdAu_3Si$ (C_{ph}) by multiplying the temperature values by this scaling factor in the C vs T curve of YAu_3Si[4]. In Figure S16, we plot the estimated C_{ph} curve with the specific heat data of $GdAu_3Si$. Note that we slightly adjust the value of C_{ph} by multiplying C_{ph} by a factor of 0.99. We estimate the magnetic contribution to the specific heat (C_{mag}) of $GdAu_3Si$ by subtracting C_{ph} from its specific heat [see Figure 8(c) in the main text]. We calculate the magnetic entropy above 0.2 K, i.e., $\Delta S_{\mathrm{mag}}(T) \equiv S_{\mathrm{mag}}(T) - S_{\mathrm{mag}}(0.2\ \mathrm{K}) = \int_{0.2\ \mathrm{K}}^{T} (C_{\mathrm{mag}}/T)\, dT$: see Figure 8(d) in the main text.

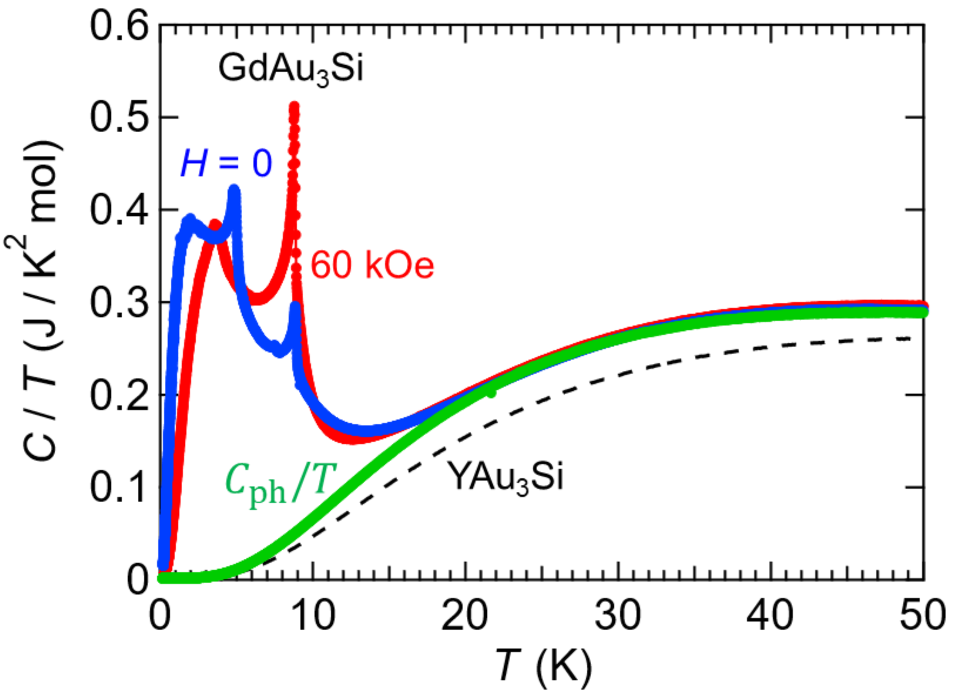

Fig. S16. The specific heat of $GdAu_3Si$ (for $H = 0$ and 60 kOe) plotted with that of nonmagnetic YAu_3Si (the broken curve) and the estimated phonon contribution of $GdAu_3Si$ (C_{ph}/T). Note that 'mol' indicates the mole of $RE_{0.2}Au_{0.6}Si_{0.2}$ (where RE = Gd, Y).

Analysis of the critical behavior of specific heat of GdAu$_3$Si

We analyze the critical behavior of the specific heat of GdAu$_3$Si at the magnetic transition at T_A. We define the normalized temperature $t \equiv (T - T_N)/T_N$, where T_N is a Néel temperature determined below. We assume the following minimal model curve in the vicinity of $t = 0$:

$$C = A_{\pm}|t|^{-\alpha} + B + Lt$$

where $\alpha, A_{\pm}, B, L$ are adjustable parameters. Note that we did not include a correction factor which is generally considered[5]. Subscripts + and – indicate $T > T_N$ and $T < T_N$, respectively. The first term describes the critical behavior, while the last two terms represent the background contribution. Figure S17(a) shows the t dependence of the specific heat of GdAu$_3$Si for $H = 0$. The experimental data are fitted to the above equation. For the fitting, we excluded a range in the vicinity of $t = 0$ (the green symbols). The experimental data (the blue symbols) are simultaneously fitted for $T > T_N$ and $T < T_N$ with a least-square method. The fitting results (with the 95% confidence bounds) are presented in the figure. We also analyze the specific-heat data for $H = 5$ kOe [see Figure S17(b)] and $H = 60$ kOe [see Figure S17(c)]. Both analytical results for $H = 0$ and $H = 5$ kOe show similar values of the critical exponent α and A_+/A_-, while the analyzed values for $H = 60$ kOe are slightly different from those of the low-field ones.

For the above fitting analysis, we set $T_N = 8.905$ K for $H = 0$, $T_N = 8.85$ K for $H = 5$ kOe and $T_N = 8.82$ K for $H = 60$ kOe. In order to determine these T_N values, we checked how the root-mean-square-error (RMSE) value of the fitting changes as the T_N value changes. Figures S17(d)-(f) show the RMSE value plotted against T_N. In the present analysis, we have determined the T_N values from the temperature exhibiting the smallest RMSE.

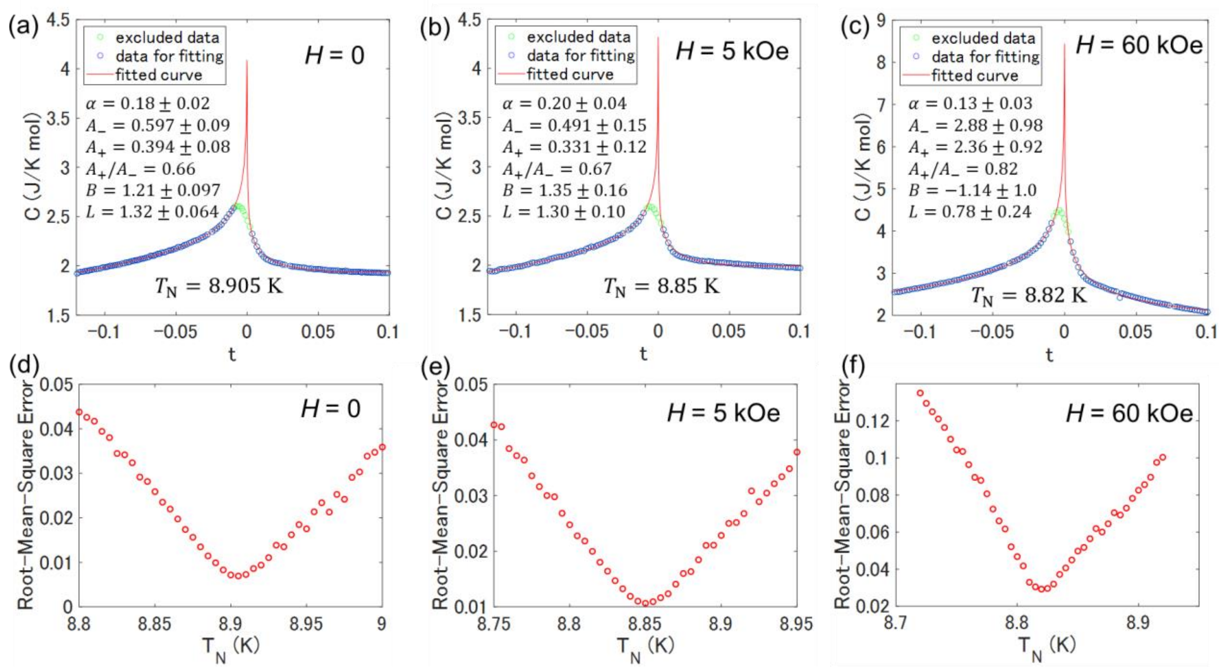

Fig. S17. (a)-(c) Specific heat plotted against the reduced temperature $t = (T - T_N)/T_N$ under the magnetic fields of (a) $H = 0$, (b) $H = 5$ kOe and (c) $H = 60$ kOe. (d)-(f) The T_N dependence of the root-mean-square-error values of the fitting for (d) $H = 0$, (e) $H = 5$ kOe and (f) $H = 60$ kOe.

References

1. Shiino, T.; Gebresenbut, G. H.; Denoel, F.; Mathieu, R.; Häussermann, U.; Rydh, A., Superconductivity at 1 K in Y-Au-Si quasicrystal approximants. *Physical Review B* **2021,** *103* (5), 054510.
2. Carbotte, J., Properties of boson-exchange superconductors. *Reviews of Modern Physics* **1990,** *62* (4), 1027.
3. Gebresenbut, G.; Shiino, T.; Eklöf, D.; Joshi, D. C.; Denoel, F.; Mathieu, R.; Häussermann, U.; Pay Gómez, C., Atomic-Scale Tuning of Tsai-Type Clusters in RE–Au–Si Systems (RE= Gd, Tb, Ho). *Inorganic chemistry* **2020,** *59* (13), 9152-9162.
4. Bouvier, M.; Lethuillier, P.; Schmitt, D., Specific heat in some gadolinium compounds. I. Experimental. *Physical Review B* **1991,** *43* (16), 13137.
5. Oleaga, A.; Salazar, A.; Prabhakaran, D.; Cheng, J.-G.; Zhou, J.-S., Critical behavior of the paramagnetic to antiferromagnetic transition in orthorhombic and hexagonal phases of $RMnO_3$ (R = Sm, Tb, Dy, Ho, Er, Tm, Yb, Lu, Y). *Physical Review B* **2012,** *85* (18), 184425.
